# Intermediate steps in the formation of neuronal SNARE complexes

**DOI:** 10.1016/j.jbc.2024.107591

**Published:** 2024-07-19

**Authors:** Sonja Pribicevic, Abigail C. Graham, David S. Cafiso, Ángel Pérez-Lara, Reinhard Jahn

**Affiliations:** 1 Laboratory of Neurobiology, Max-Planck Institute for Multidisciplinary Sciences, Göttingen, Germany; 2 Department of Chemistry, University of Virginia, Charlottesville, Virginia, USA; 3 Department of Physical Chemistry, Faculty of Pharmacy, University of Granada, Granada, Spain

**Keywords:** SNARE proteins, pre-steady-state kinetics, fluorescence resonance energy transfer (FRET), electron paramagnetic resonance (EPR), double electron-electron resonance (DEER), fusion protein, complex assembly

## Abstract

Neuronal exocytosis requires the assembly of three SNARE proteins, syntaxin and SNAP25 on the plasma membrane and synaptobrevin on the vesicle membrane. However, the precise steps in this process and the points at which assembly and fusion are controlled by regulatory proteins are unclear. In the present work, we examine the kinetics and intermediate states during SNARE assembly *in vitro* using a combination of time resolved fluorescence and EPR spectroscopy. We show that syntaxin rapidly forms a dimer prior to forming the kinetically stable 2:1 syntaxin:SNAP25 complex and that the 2:1 complex is not diminished by the presence of excess SNAP25. Moreover, the 2:1 complex is temperature-dependent with a reduced concentration at 37 °C. The two segments of SNAP25 behave differently. The N-terminal SN1 segment of SNAP25 exhibits a pronounced increase in backbone ordering from the N- to the C-terminus that is not seen in the C-terminal SNAP25 segment SN2. Both the SN1 and SN2 segments of SNAP25 will assemble with syntaxin; however, while the association of the SN1 segment with syntaxin produces a stable 2:2 (SN1:syntaxin) complex, the complex formed between SN2 and syntaxin is largely disordered. Synaptobrevin fails to bind syntaxin alone but will associate with syntaxin in the presence of either the SN1 or SN2 segments; however, the synaptobrevin:syntaxin:SN2 complex remains disordered. Taken together, these data suggest that synaptobrevin and syntaxin do not assemble in the absence of SNAP25 and that the SN2 segment of SNAP25 is the last to enter the SNARE complex.

Fusion of synaptic vesicles with the plasma membrane is one of the key events in neuronal signaling. It is tightly controlled by a multitude of proteins, with the soluble N-ethylmaleimide-sensitive factor (NSF) attachment protein receptors (SNAREs) being responsible for catalyzing the merger of the bilayers. SNARE proteins contain conserved stretches of 60 to 70 amino acids with heptad repeats termed SNARE motifs. They are highly conserved during evolution and are divided into four subfamilies, referred to as Qa, Qb, Qc, and R SNAREs (1).

The SNARE motifs of the neuronal SNAREs, syntaxin-1A (Qa), SNAP25 (Qbc), and synaptobrevin 2 (R), are mainly unstructured as monomers (2). Upon interaction, they form a four-helix bundle of extraordinary stability (3, 4) that requires ATP, AAA-ATPase NSF, and the adaptor proteins α-SNAPs for its disassembly (5). The energy released during formation of the SNARE complex is thought to directly translate into deformation of the membranes, which leads to their fusion (6).

A long-standing problem has been that using SNAREs only, in-vitro assembly, while it ultimately ends up in the stable ternary complex, is agonizingly slow, particularly for the neuronal SNARE complex, requiring many minutes to hours for completion (7). This is true for soluble and for membrane-anchored SNAREs, as fusion of liposomes containing only neuronal SNARE proteins proceeds with extremely slow kinetics (8), orders of magnitude slower than neuronal exocytosis (9). This is probably caused by the propensity of the three SNAREs to assemble in multiple compositions and stoichiometries, many of which probably represent kinetically trapped “off-pathway” complexes (see below), requiring regulatory proteins to stabilize the intermediate states of the assembly pathway. Indeed, since the original observations, SNARE-mediated fusion has been reconstituted with sub-second kinetics using either co-assembly with regulatory proteins including the neuronal calcium sensor synaptotagmin (10, 11, 12) or using artificial stabilization of intermediate acceptor complexes (*e.g.* (13, 14)).

Despite such progress, it is still debated in which sequence the four SNARE motifs (two of which are connected in SNAP25) bind to each other to arrive at the QabcR four-helix bundle. Detailed studies have revealed that SNARE proteins spontaneously assemble in various combinations, with most studies dealing with SNARE assembly in solution using constructs lacking the membrane anchors (see (15)). For instance, the Q-SNARE motifs readily form a 2:1 four-helix bundle (Qaabc; *e.g.* (2, 16, 17, 18)). Other binary complexes have also been reported such as Qaabb (19), QaR (*e.g.* (20, 21, 22)), or QbcR (15, 23), but they are less well characterized and may require special conditions for stabilization. Additionally, syntaxin has a tendency to form well-structured oligomers including two- and four-helix Qa-bundles (24, 25). Furthermore, it is well known that syntaxin-1A can fluctuate between an “open” and “closed” conformation influencing its ability to bind other SNAREs (26, 27, 28). All these complexes form thermodynamic minima in the assembly landscape, and it is unclear which of them serves as an intermediate in the assembly pathway and which represent side-reactions that render the SNAREs unable to proceed to the formation of the QabcR SNARE complex.

Presently, it is widely accepted that regulatory proteins including Munc-18, Munc-13, complexin, and synaptotagmin guide SNAREs through the assembly pathway (29), thus preventing them from being trapped in nonproductive side reactions. It is however uncertain whether regulatory proteins stabilize pre-existing acceptor SNARE complexes that are of low abundance or whether they act as scaffolds to provide independent binding sites for SNARE motifs that will not assemble until other critical SNARE motifs are present. This is particularly important for the final step: the insertion of the last SNARE motif into a structured preformed acceptor complex, which results in SNARE zippering and thus constitutes the “power stroke” for membrane fusion (30, 31).

For these reasons, we have re-investigated the kinetics of the various assembly reactions that neuronal SNARE proteins can undergo in the absence of regulatory proteins, expanding on previous work by this and other laboratories (*e.g.* (2, 20, 22, 32, 33)). Our goal was to understand the different binding equilibria and to identify the steps that may serve as intermediates in the pathway towards formation of the QabcR complex, which are acted upon by regulatory proteins. To this end, we carried out stopped-flow titrations of the SNAREs in various combinations using fluorescence resonance energy transfer (FRET), complemented by more in-depth analysis of assemblies by continuous wave electron paramagnetic resonance (CW EPR) and double electron-electron resonance (DEER) spectroscopy.

## Results

### Syntaxin interacts with SNAP25 as a dimer

The starting point of our investigation was to measure the kinetics of the binary interaction between syntaxin and SNAP25. We labeled the proteins with the fluorescent dyes Alexa Fluor 488 (donor) or Alexa Fluor 647 (acceptor) as indicated and monitored binding by FRET using a kinetic assay capable of detecting small and rapid changes. In this and all following experiments, we used variants of syntaxin, SNAP25, and synaptobrevin in which all endogenous cysteines were replaced by serine, and single cysteines were introduced in the middle regions of the SNARE motifs for labeling (syntaxin-1A, 1–265, S225C; SNAP25a, 1–206, M49C; synaptobrevin 2, 1–96, S61C). These mutations were previously shown not to hinder complex formation (7, 17) and in our experiments showed a robust FRET signal upon assembly.

**Figure 1:**
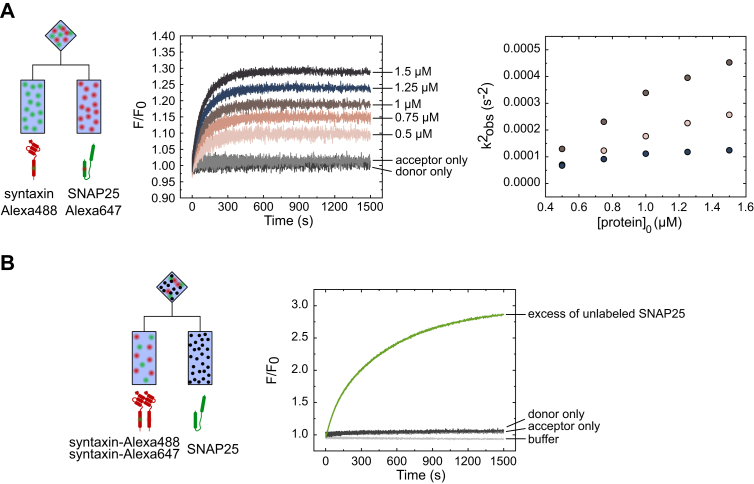
**Syntaxin and SNAP25 form a 2:1 complex.***A*, equimolar titration of syntaxin labeled with Alexa Fluor 488 (donor, *green* dots) and SNAP25 labeled with Alexa Fluor 647 (acceptor, *red dots*) using a stopped-flow setup equilibrated at 37 °C (scheme on the *left*). The donor was excited using a 470 nm LED lamp, and the change in the acceptor fluorescence was monitored over time. A robust, concentration-dependent FRET signal was observable. *Right*: plot of k^2^_obs_ against the initial concentration of either syntaxin or SNAP25. Note the high variability between independent experiments and a nonlinear (hyperbolic, Fig. S1, *C*–*E*) increase in the k^2^_obs_ with the increasing protein concentrations. Different colors in the scatter plot represent three biological replicates of the titration experiment. *B*, addition of excess SNAP25 (7.5 μM) to a mixture of syntaxin labeled with donor or the acceptor dye (1.5 μM final, 0.75 μM for each labeled variant). A strong increase in FRET signal was observed indicating the formation of a 2:1 syntaxin:SNAP25 complex. F/F_0_ - ratio between the measured fluorescence intensity and the average fluorescence intensity at the onset of the reaction (first 100 data points); acceptor only – control in which the donor dye is omitted; donor only – control in which the acceptor dye is omitted; buffer – control in which the unlabeled protein was omitted.

We titrated syntaxin and SNAP25 at 1:1 M ratio (Fig. 1*A*, left panel), resulting in a concentration-dependent increase of the FRET signal (Fig. 1*A*, middle panel and Fig. S1*B*). The plot of the square of the observed rate constant (k_obs_^2^) *versus* the initial concentration of either syntaxin or SNAP25 showed a hyperbolic increase (Figs. 1*A*, right panel and S1, *C*–*E*), suggesting that SNAP25 and syntaxin interaction does not occur in a 1:1 stoichiometry. Moreover, analysis by size-exclusion chromatography of the reaction product after overnight incubation at 4 °C revealed an excess of free SNAP25 (Fig. S1*A*). Both observations are consistent with the preferential formation of a complex containing two syntaxin and one SNAP25 molecules (2:1 complex), which was expected based on earlier studies (2, 17, 18).

Previous work has shown that the 2:1 complex is composed of a four-helix bundle that resembles the fully assembled SNARE complex, with the position of the R-SNARE synaptobrevin being occupied by a second syntaxin molecule (17, 18, 34). It is generally considered to represent an “off-pathway” complex that forms stepwise, with binding of the first syntaxin molecule preceding that of the second (7, 13, 33). Accordingly, adding excessive concentrations of SNAP25 is predicted to shift the equilibrium to a 1:1 complex, with SNAP25 capturing all syntaxin molecules during the first binding step (13, 35). However, when a 10-fold excess of SNAP25 was added to an equimolar mix of syntaxin molecules labeled with either the donor or the acceptor dye, respectively (Fig. 1*B*, left panel), a major increase in the acceptor fluorescence was observed. (Fig. 1*B*, right panel).

**Figure 2:**
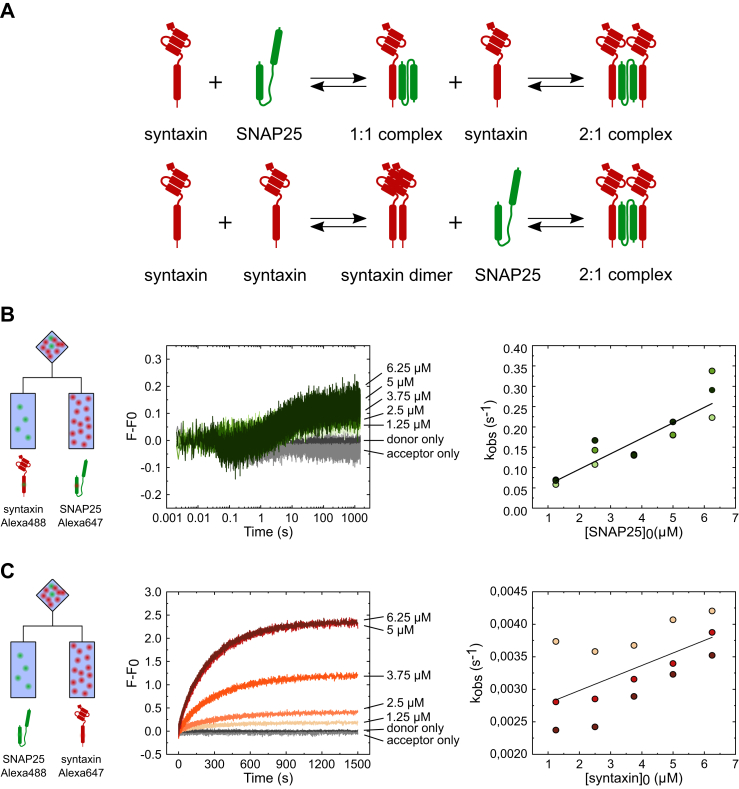
**The “syntaxin-dimer first” model better explains formation of the 2:1 complex.***A*, schematic overview over two alternative assembly pathways for the formation of the 2:1 complex: “1:1 complex first” (*upper panel*) and “syntaxin-dimer first” (*lower panel*). *B*, titration by stopped-flow of increasing concentrations of acceptor-labeled SNAP25 over a fixed concentration (0.125 μM) of donor-labeled syntaxin (see Fig. 1 for color-coding of donor and acceptor dyes in the cartoons). Note that FRET increase was fast but exhibited only a low maximal amplitude that barely changed with increasing SNAP25 concentrations. *Right*: the plot of k_obs_ over the initial SNAP25 concentration ([SNAP25]_0_) shows a linear increase with a slope of (3.8 ± 0.3) × 10^−2^ and an intercept of (1.9 ± 0.9) × 10^−2^. *C*, titration of increasing concentrations of acceptor-labeled syntaxin over a fixed concentration (0.125 μM) of donor-labeled SNAP25. In contrast to (*B*), a major increase of FRET was observed that increased with increasing syntaxin concentration. *Right*: the plot of k_obs_ over the initial syntaxin concentration ([syntaxin]_0_) showed a linear increase with a slope of (1.61 ± 0.29) × 10^−4^ and an intercept of (3.2 ± 0.1) × 10^−3^. Different colors in the scatter plots represent three biological replicates of the titration experiments.

To explain this unexpected observation, we considered an alternative pathway for assembly that was suggested earlier (25) but not followed up later. Accordingly, syntaxin first dimerizes and then binds as a dimer to SNAP25 (see the cartoon in Fig. 2*A*). Indeed, previous studies have shown that the SNARE motifs of syntaxin tend to oligomerize both in solution (with a Kd of 1.4 ± 0.5 μM for the monomer-dimer and of 12 ± 6 μM for the dimer-tetramer equilibrium, measured at 4 °C; (24, 25)), as well as in membranes where oligomerization results in the formation of clusters (36, 37, 38). Accordingly, binding of syntaxin dimers to SNAP25 would deplete the free dimers, resulting in the formation of more dimers by mass-action that are then captured by SNAP25 in the 2:1 complex until all syntaxin is consumed, explaining the increase in FRET.

To distinguish between the two possible assembly pathways, we performed two complementary titration experiments. First, we titrated acceptor-labeled SNAP25 over donor-labeled syntaxin (Fig. 2*B*, left panel). This experiment showed a rapid increase in the acceptor fluorescence, but the amplitude was low and did not increase with increasing SNAP25 concentration (Fig. 2*B*, middle panel). The titration traces were, with some difficulty due to the high noise, fit to a monoexponential equation (Fig. S2*A*) to obtain k_obs_, which showed a linear dependence on the initial SNAP25 concentration (Fig. 2*B*, right panel).

In contrast, titration of acceptor-labeled syntaxin over donor-labeled SNAP25 resulted in traces with much higher amplitudes that increased with the syntaxin concentrations (Fig. 2*C*, left and middle panel). The traces could be easily fit to a monoexponential equation (Fig. S2*B*), with k_obs_ also showing a linear dependence on the initial concentration of syntaxin (Fig. 2*C*, right panel).

The remarkable difference between these two experiments (see Fig. S2, *C* and *D*) supports the “dimer first” model rather than the “1:1 complex first” model, for the following reasons. If a syntaxin dimer needs to form first, formation of the 2:1 complex will be limited by the equilibrium concentration of the dimer, which is predicted to be low at an overall syntaxin concentration of 0.125 μM: when taking into consideration the previously published Kd of 1.4 ± 0.5 μM for dimer formation measured at 4 °C (24), the concentrations of monomer and dimer should be ∼0.11 μM and ∼0.01 μM, respectively. Given that Kd is temperature-dependent, the concentration of dimer should be even lower considering that our kinetic experiments were performed at 37 °C, thus explaining why even in the presence of excess SNAP25, the steady-state concentration of the 2:1 complex remains low. In contrast, increasing the overall syntaxin concentration causes an increase in dimer concentration, which reacts with SNAP25, resulting in major increase of the amplitude of the FRET signal, as seen in Figure 2*C*.

**Figure 3:**
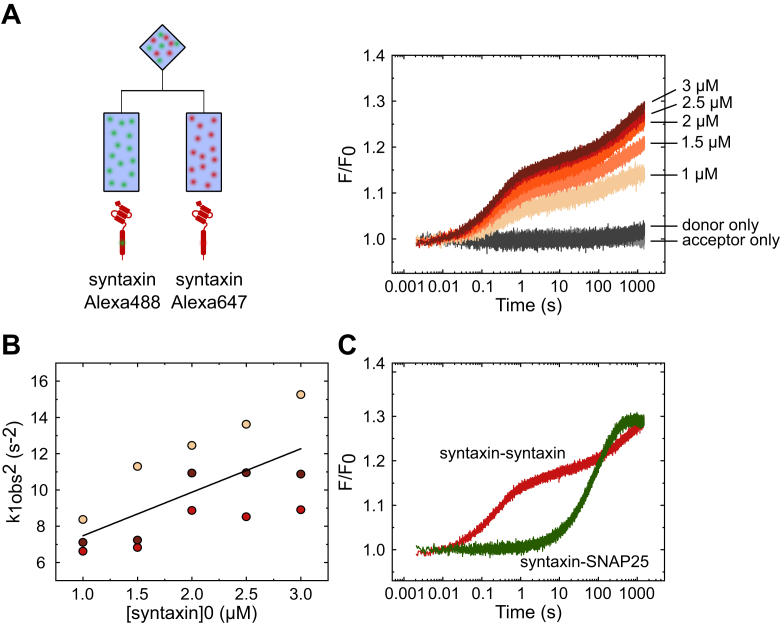
**Syntaxin dimerization is faster than syntaxin–SNAP25 interaction.***A*, titration by stopped-flow of equimolar concentrations of syntaxin labeled either with Alexa Fluor 488 (donor) or Alexa Fluor 647 (acceptor). The increase in the acceptor fluorescence revealed complex kinetics, with a fast initial phase that increased with increasing syntaxin concentration. *B*, the obtained k_1obs_ values were squared and plotted over the initial syntaxin concentration ([syntaxin]_0_, sum of both donor- and acceptor-labeled fractions). The linear dependence between the k_1obs_^2^ and [syntaxin]_0_ yielded an average on-rate of ∼ (1.3 ± 0.2) × 10^5^ M^−1^s^−1^ and an average off-rate of 2.2 ± 0.1 s^−1^. The rate constants were obtained using the following linear formula: k_obs_^2^ = 8k_1_k_-1_[syntaxin]_0_ + (k_-1_)^2^, where 8k_1_k_-1_ is the slope and where (k_-1_)^2^ is the intercept (41). *C*, comparing the kinetics of syntaxin dimerization with the equivalent kinetics of syntaxin-SNAP25 binding showed that syntaxin-syntaxin binding is faster than syntaxin-SNAP25 binding. Different colors in the scatter plot represent three biological replicates of the titration experiment.

To better understand how the formation of syntaxin oligomers determines its interactions with SNAP25, we measured the kinetics of syntaxin oligomerization under our experimental conditions. To this end, we labeled syntaxin with either the donor or the acceptor dye and performed equimolar titrations similar to those described above for syntaxin and SNAP25 (Figs. 1*A*, and 3*A*). As shown in Figure 3*A* (right panel) and Fig. S3, the FRET traces revealed several kinetically distinct phases, but only the first phase, presumably corresponding to the dimer formation, showed dependence on the concentration of syntaxin (Fig. 3*B*). Remarkably, syntaxin dimerization was much faster at all concentrations than the formation of the 2:1 complex that was measured under identical conditions (comparison of the highest concentration shown in Fig. 3*C*) and thus is not rate-limiting.

To confirm that the concentration of the 2:1 complex is very low under equilibrium conditions, we carried out “displacement” experiments in which we allowed the 2:1 complex to form using labeled proteins and then added a 10-fold excess of unlabeled SNAP25 or syntaxin, respectively (Fig. S4, *A* and *E*). As expected, addition of SNAP25 resulted in a decrease (Fig. S4, *B* and *C*). In contrast, addition of unlabeled syntaxin resulted in an increase (Fig. S4*F*), suggesting that even at this excessive concentration (7.5 μM), the formation of the 2:1 complex is still dominant (see above, Fig. 2*C*). Taken together, these data indicate that the starting concentration of the 2:1 complex is very low due to the low steady-state concentrations of the syntaxin dimer, with SNAP25 only being saturated at excessive syntaxin concentrations.

### Formation of the syntaxin:SNAP25 2:1 complex is strongly temperature-dependent

**Figure 4:**
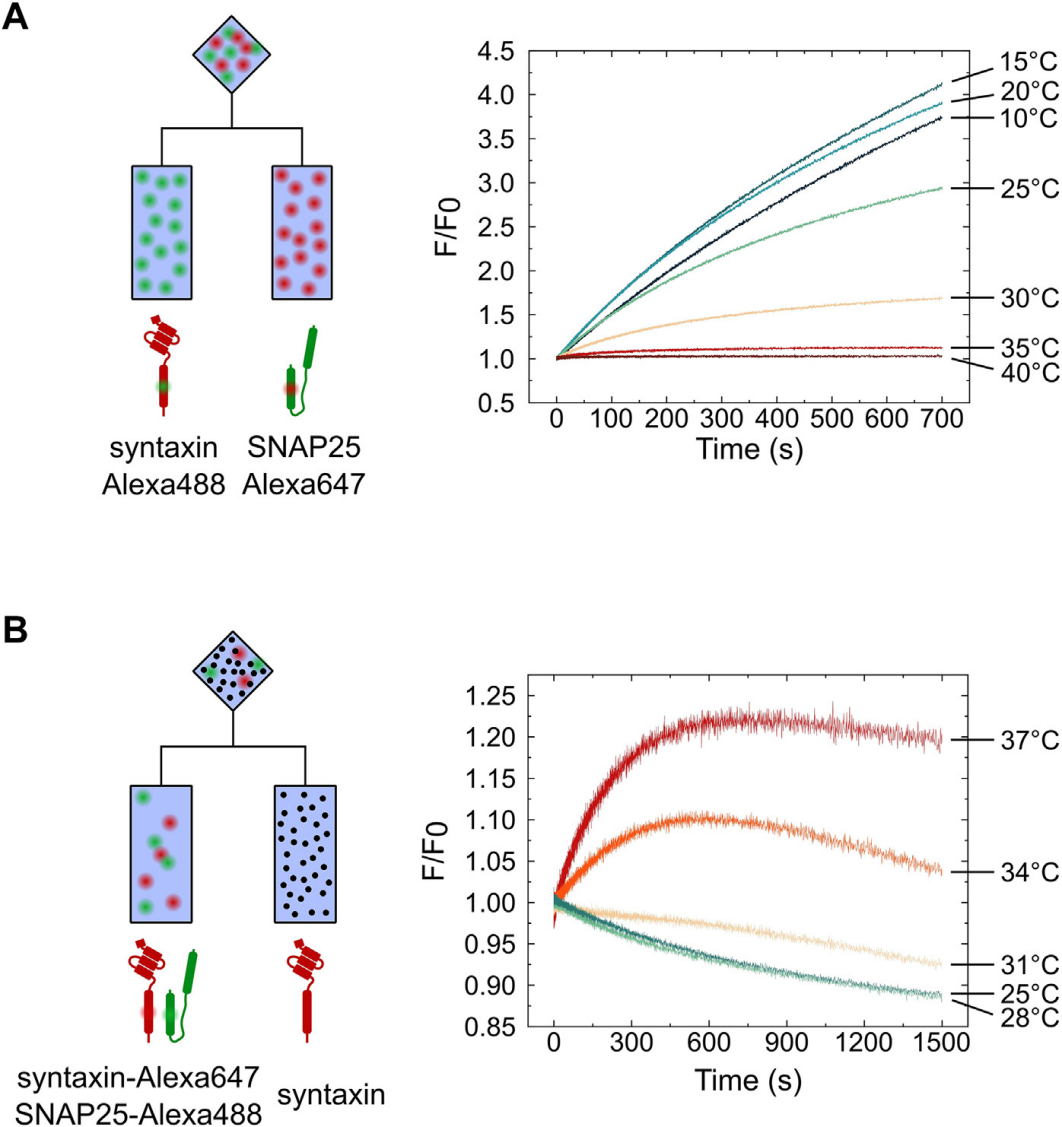
**Formation of the 2:1 complex strongly depends on the temperature.***A*, formation of the 2:1 complex at different temperatures (initial concentration of 0.75 μM for both syntaxin and SNAP25 at all temperatures). The amplitude of the FRET signal shows a striking increase with decreasing temperature, reaching saturation at ∼15 °C (Fig. S5*C*). *B*, addition of excess unlabeled syntaxin (7.5 μM) to a pre-incubated mix of donor-labeled SNAP25 and acceptor-labeled syntaxin at different temperatures. The FRET signal strongly increases at physiological temperatures but decreases already at 31 °C and lower temperatures.

The low efficiency in forming the syntaxin–SNAP25 complex observed in our kinetic experiments is difficult to reconcile with previous reports (including this study; Fig. S1*A*) where high yields of the 2:1 complex were obtained which were sufficiently stable to allow for its separation from free syntaxin and SNAP25. We therefore tested whether these differences may be due to different temperatures used in the assembly experiments. All of our kinetic experiments were performed at 37 °C, in contrast to most previous studies where lower temperatures were used (usually 25 °C or 4 °C; *e.g.* (2, 7, 30, 33, 39)). We thus repeated the assembly experiment shown in Figure 1*A*, using equimolar (0.75 μM) concentrations of donor-labeled syntaxin and acceptor-labeled SNAP25 at different temperatures (Fig. 4*A*). While the reaction rate increased with the temperature (reflected in the curvature of the traces and the speed with which the plateau was reached; Fig. S5, *A* and *B*), we observed a rather dramatic decline of the equilibrium signal (Fig. 4*A*, right panel; Fig. S5*C*), indicating that at 37 °C, the concentration of the 2:1 complex is very low. We then repeated our "displacement" experiments in conditions of 10-fold excess of unlabeled syntaxin (Figs. 4*B* and S4, *E* and *F*). When normalized to the equilibrium signal, the FRET signal showed again an increase at 37 °C. In contrast, a decrease was observed at temperatures below 30 °C showing that the excess of unlabeled syntaxin is now able to compete with the labeled syntaxin and displace it from the 2:1 complex (Fig. 4*B*), corroborating higher starting concentrations of the 2:1 complex at lower temperatures. This data shows that the formation of the 2:1 complex is strongly dependent on the temperature with a very low equilibrium concentration at 37 °C.

### Assembly of synaptobrevin with other SNAREs requires the formation of a syntaxin–SNAP25 complex

As outlined in the Introduction, the sequence of steps in the formation of the ternary SNARE complex is still controversial. Whereas most of the earlier work suggested that synaptobrevin can only bind after some assembly between SNAP25 and syntaxin (*e.g.* (7, 33, 40)), an alternative hypothesis has recently gained favor stating that syntaxin and synaptobrevin need to be aligned before SNAP25 can bind, with the alignment being facilitated by simultaneous binding to the SM-protein Munc18 (reviewed in (29)). In contrast to the syntaxin–SNAP25 interaction that has been well documented in many publications, it is unclear how stable and abundant the reported binary interactions between synaptobrevin and either syntaxin or SNAP25 (*e.g.* (22, 23)) are in the absence of the SM-protein Munc18.

Using fluorescently labeled synaptobrevin, we therefore checked whether the protein undergoes binary interactions with either syntaxin or SNAP25 in our experimental conditions, but no change in the FRET signals was observable even at high concentrations (Fig. S6, *A* and *C*). Similarly, no evidence for binary complexes was obtained when the proteins were pre-incubated and then analyzed by size-exclusion chromatography (Fig. S6, *B* and *D*), in line with most previous reports. Moreover, no evidence for interactions between synaptobrevin and syntaxin or either of the SNARE motifs of SNAP25 was obtained by electron paramagnetic resonance (EPR) spectroscopy. In these experiments, synaptobrevin was spin-labeled at different positions along the SNARE motif to capture interactions that might have escaped detection by FRET (Fig. S11, *D* and *E*). As we saw no evidence for either syntaxin–synaptobrevin or SNAP25–synaptobrevin complexes, we concentrated our study on the binding of synaptobrevin to syntaxin–SNAP25 complexes.

To this aim, we carried out titration experiments with all three SNARE proteins using donor-labeled syntaxin and acceptor-labeled SNAP25, while the unlabeled synaptobrevin was titrated in excess. The labeled proteins were in 1:1 M ratio, and their concentration did not change between different titration steps, meaning that any observed change originates from the changing concentration of the unlabeled titrant.

**Figure 5:**
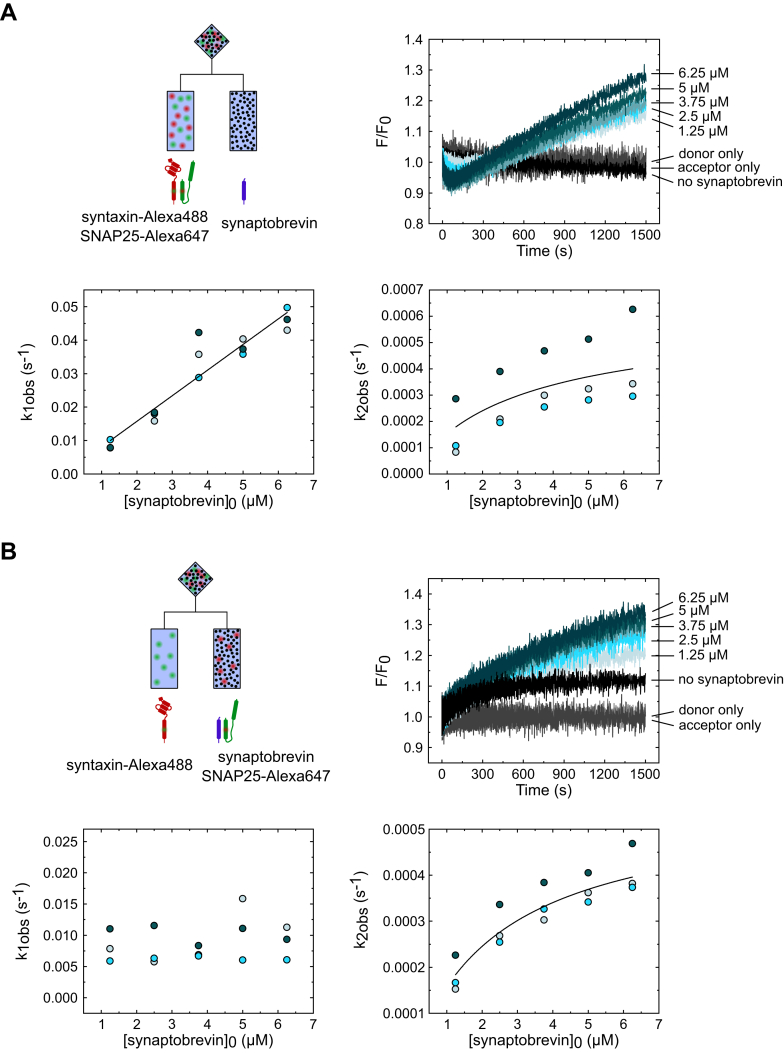
**Synaptobrevin titration experiments confirm syntaxin–SNAP25 interaction as the first step in the SNARE assembly reaction.** Titration experiments were performed in two ways: (*A*) synaptobrevin was added to the premixed syntaxin and SNAP25 or (*B*) SNAP25 and synaptobrevin were added at the same time to syntaxin. Two phases, exponential and linear, could be distinguished in the titration traces of both experiments. *A*, in the case of preformed 2:1 complex, addition of excess synaptobrevin caused an initial dip in the fluorescence signal that was followed by a steady liner increase. The apparent rate of the fluorescence dip depended on synaptobrevin concentration revealing an irreversible step with a rate constant of (7520 ± 200) M^−1^s^−1^ that presumably corresponds to the displacement of the second syntaxin from the 2:1 complex. *B*, when synaptobrevin was added at the same time as SNAP25, the first exponential phase did not change with synaptobrevin concentration, indicating interaction between syntaxin and SNAP25 only. This was confirmed with “no synaptobrevin” control (*black* trace) that showed the same exponential increase which also corresponded to the equimolar syntaxin-SNAP25 titration (see Fig. 1). The linear phase of both experiments showed a hyperbolic dependence on synaptobrevin concentration as expected, given that the rate of synaptobrevin binding is limited by the rate of the 2:1 complex formation. Different shades of blue in the scatter plots represent three biological replicates of the titration experiments.

Considering that our results so far all confirmed that syntaxin and SNAP25 form a 2:1 complex, synaptobrevin titration was performed in two ways: (1) syntaxin and SNAP25 were premixed to allow the 2:1 complex to equilibrate before the addition of synaptobrevin (Fig. 5*A*); (2) SNAP25 was premixed with synaptobrevin and added to syntaxin at the same time (Fig. 5*B*), with no 2:1 complex being present at the start of the reaction. In both cases, fluorescence traces displayed two phases: an exponential phase followed by a linear one, suggesting at least two steps with widely separated rate constants (41).

In the case of premixed syntaxin and SNAP25, the fluorescence traces displayed an initial dip that was followed by a steady linear increase (Fig. 5*A*, upper right panel; Fig. S9*A*), with observed rate constants of both phases (k_1obs_, k_2obs_) dependent on synaptobrevin concentration (Fig. 5*A*, lower panels) In contrast, addition of constant amount of SNAP25 premixed with increasing concentration of synaptobrevin to syntaxin (Figs. 5*B* and S9*B*) showed an initial fast, but relatively small fluorescence increase, followed again by a steady and larger linear increase. This time, the observed rate constants of the initial fast phase (k_1obs_) changed independently of synaptobrevin concentration (Fig. 5*B*, lower left panel), while the slower linear phase showed dependence of synaptobrevin concentration similar to that observed above (Fig. 5*B*, lower right panel).

The conspicuous differences between the fast phases are best explained by a reaction sequence in which the 2:1 complex needs to form as an intermediate before one of the syntaxins is being displaced by synaptobrevin. When SNAP25 is added together with synaptobrevin, the initial fast formation of the 2:1 complex is followed by a dynamic equilibrium in which its steady-state concentration is determined by its (fast) formation and (slow) consumption, with formation being independent of the synaptobrevin concentration. When the 2:1 complex is allowed to reach equilibrium before addition of synaptobrevin, the displacement by synaptobrevin of one of the syntaxins determines the initial fast phase (therefore it is dependent on the synaptobrevin concentration), explaining the transient drop in FRET, until the same dynamic equilibrium as above is reached. In both cases, the relatively small amplitudes of the fast phase are due to the low equilibrium concentration of the 2:1 complex at 37 °C (see above), while the slow linear phase corresponds to the final and virtually irreversible assembly of the ternary SNARE complex.

In addition to the titration of unlabeled synaptobrevin, titrations of unlabeled SNAP25 and syntaxin in excess were also performed and can be found in Figs. S7 and S8, respectively. These data are in agreement with synaptobrevin titration experiments in showing that the syntaxin–SNAP25 2:1 complex is prerequisite for synaptobrevin binding.

### The two SNARE motifs of SNAP25 (SN1 and SN2) are primarily disordered in solution, but SN1 exhibits some backbone order towards the N-terminus

Unlike syntaxin and synaptobrevin, SNAP25 contributes two motifs to the SNARE complex, SN1 (Qb) at its N terminus and SN2 (Qc) at its C terminus (4); both motifs contribute an alpha-helix to the ternary 2:1 complex as well (18). However, it is unclear whether both segments have a similar behavior and incorporate at the same point during SNARE assembly (42). To better understand the properties of SN1 and SN2 and their contributions during assembly with syntaxin and synaptobrevin, we examined the backbone dynamics and conformational landscape of the individually purified SN1 and SN2 fragments using a combination of CW EPR spectroscopy and DEER.

**Figure 6:**
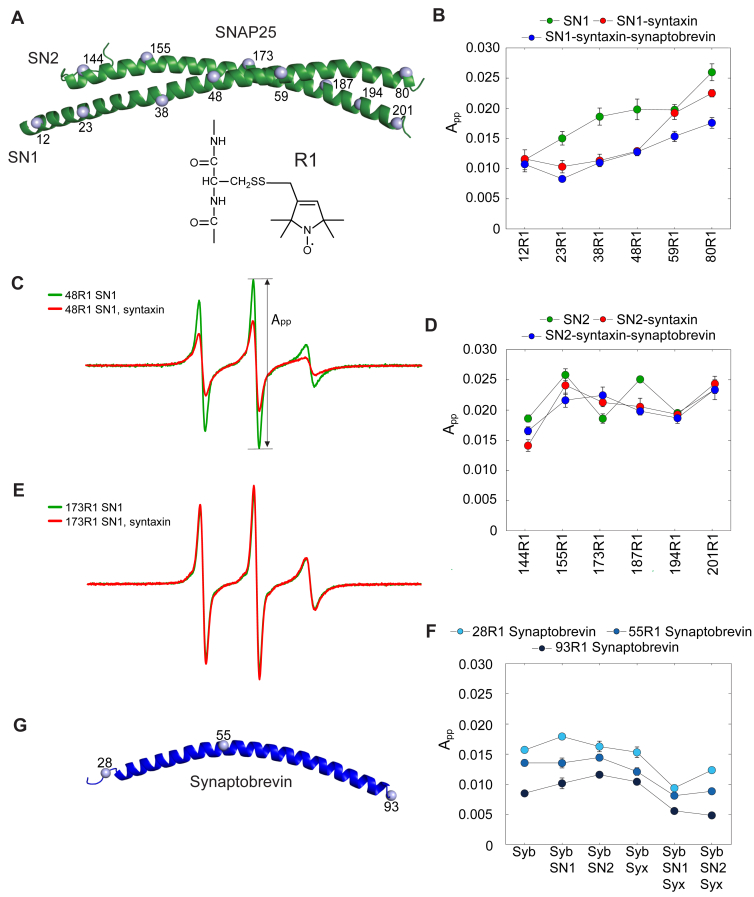
**SN1 and SN2 interact differently with syntaxin and synaptobrevin.***A*, SNAP25 (from PDB ID: 1SFC) showing the α-carbons of the 12 spin labeled positions as *gray spheres*. The nitroxide side chain R1 is incorporated into six positions on SN1 and SN2 by reacting engineered cysteine side chains with an MTS reagent (see Experimental procedures). *B*, the normalized average peak-to-peak amplitude of the centerline of the EPR spectra (A_pp_) as a function of position along SN1. The value of (A_pp_) provides a relative measure of label motion (65) and is related here to the mobility of the SN1 backbone. Data is shown for isolated SNAP25 SN1 at 24 μM (*green*), 24 μM SN1 with 32 μM syntaxin (*red*), and 24 μM SN1 with 32 μM syntaxin and 24 μM synaptobrevin (*blue*). *C*, normalized X-band EPR spectra of 24 μM 48R1 SNAP25 SN1 in the absence (*green*) and presence of 32 μM syntaxin (1-250) (*red*). *D*, the value of (A_pp_) obtained for the six spin labeled positions on SN2. Data is shown for isolated SNAP25 SN1 at 24 μM (*green*), 24 μM SN1 with 32 μM syntaxin (*red*), and 24 μM SN1 with 32 μM syntaxin and 24 μM synaptobrevin (*blue*). *E*, normalized X-band EPR spectra of 24 μM 173R1 SNAP25 SN2 in the absence (*green*) and presence of 32 μM syntaxin (1-250) (*red*). *F*, the value of (A_pp_) obtained for the three spin labeled positions on synaptobrevin at sites 28R1 (*cyan*), 55R1 (*blue*), and 93R1 (*black*), at concentrations of 25, 26, and 25 μM, respectively. Data are shown for synaptobrevin alone and with the addition of SN1, SN2, and syntaxin. SN1, SN2, and syntaxin are present at concentrations of 28 to 30 μM. *G*, synaptobrevin (PDB 1SFC) with the α-carbons of the R1 label positions highlighted as *gray spheres*.

To examine the local structure and mobility of the isolated SN1 and SN2 motifs, we produced six derivatives each of SN1 and SN2 where the spin-labeled side chain R1 was engineered at single positions as shown in Figure 6*A*. We then examined the EPR spectra from each site (Figs. 6, *B*–*E* and S11, *A* and *B*). EPR spectra recorded from these labeled sites yield spectra with narrow linewidths, where the label correlation times are on the order of 1 ns. These spectra are similar to those at dynamic protein sites with little defined structure (43). However, spin labels towards the N-terminal half of SN1 execute noticeably slower label motion, as indicated by their decreased normalized intensity (A_pp_) (Fig. 6*B*). At the N terminus, the correlation time for the label was about 20% longer than that observed at the C terminus, suggesting there is a reduction in backbone motion at the N terminus. This may be due to an increased helical content at this end of SN1. Compared to SN1, SN2 also yields spectra consistent with a protein backbone that is dynamic and disordered; however, unlike SN1, there is no clear gradient in motion as one proceeds from the N- to the C-terminal end of the segment (Fig. 6*D*). Thus, except for the N-terminal end of SN1, both fragments are largely unstructured in solution.

### Isolated SN1 self-associates in solution

For the isolated soluble fragments of SN1 and SN2, several single-labeled derivatives were examined using DEER. For a protein with a spin label at a single site that is randomly distributed, the background-corrected DEER data should yield no evidence for a dipole–dipole interaction with a defined distance. In this case, there should be no modulation depth in the corrected DEER signal (44). With the resonator and conditions used in these pulse EPR experiments, modulation depths are typically 0.2 to 0.25 for an intermolecular dipole interaction between two spins for a fully labeled protein.

**Figure 7:**
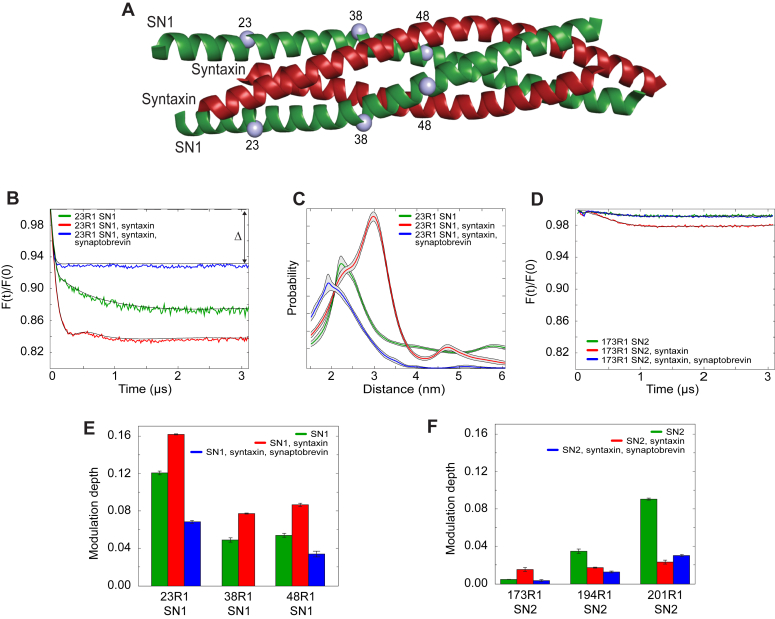
**SN1 and SN2 form complexes of different stability with syntaxin and synaptobrevin.***A*, the 2:2 complex: SN1 SNAP25 (*green*), two syntaxin (*red*) (PBD: IJTH). The *gray spheres* represent the R1 label positions along SN1. *B*, background-corrected DEER data obtained for 26 μM 23R1 SN1 (*green trace*), 26 μM 23R1 SN1 in the presence of 26 μM syntaxin (*red trace*), 26 μM 23R1 SN1 in the presence of 26 μM syntaxin and 26 μM synaptobrevin (*blue trace*). *C*, distance distributions for 26 μM 23R1 SN1 (*green*), 26 μM 23R1 SN1 in the presence of 26 μM syntaxin (*red*), and 26 μM 23R1 SN1 in the presence of 26 μM syntaxin and 26 μM synaptobrevin (*blue trace*). *D*, background-corrected DEER data obtained for 31 μM 173R1 SN2 (*green trace*), 31 μM 173R1 SN2 in the presence of 31 μM syntaxin (*red trace*), 31 μM 173R1 SN2 in the presence of 31 μM syntaxin and 31 μM synaptobrevin (*blue trace*). *E*, modulation depths (Δ), measured from background-corrected DEER data for SN1 labels 23R1, 38R1, and 48R1 when isolated (*green*), in the presence of syntaxin (*red*), and in the presence of syntaxin and synaptobrevin (*blue*). *F*, modulation depths (Δ), measured from background-corrected DEER data for SN2 labels 173R1, 194R1, and 201R1 when isolated (*green*), in the presence of syntaxin (*red*), and in the presence of syntaxin and synaptobrevin (*blue*). Bar plots represent mean ± SD.

Three single labeled sites on SN1 and three sites on SN2 (Fig. 7*A*) were examined using DEER and the results are shown in Figure 7 and in Fig. S12. Shown in Figure 7, *B* and *C* are background-corrected DEER signals and distance distributions for 23R1 on SN1, respectively. For isolated SN1, significant modulation depths are obtained at each position examined and are largest at position 23 near the N terminus (Fig. 7*E*). At this site, the distribution is broad, indicating that the structure formed is heterogeneous, although the dominant distance in this distribution near 23 Å is close to that expected for two labels interacting across a helical dimer. From these data, the presence of higher order oligomers cannot be ruled out, but the data demonstrate that isolated SN1 is capable of self-association. For SN2, the modulation depths are much weaker (Fig. 7*D*) and are insignificant towards the middle at position 173 (Fig. 7*F*). Self-association of SN2 appears to be strongest towards the C terminus (Fig. 7*F*).

### SN1 (Qb) and SN2 (Qc) interact differently with syntaxin and synaptobrevin

First, labeled SN1 was combined with WT SN2 to determine if SN1 and SN2 bind to each other in the absence of syntaxin and synaptobrevin. No change in the normalized intensities along the SN1 backbone were observed, indicating these two SNAP25 motifs do not bind to one another through a stable interaction which induces additional backbone ordering in SN1 (Fig. S11*C*). Next, structural changes in SN1 and SN2 were examined in the presence of syntaxin and synaptobrevin. When syntaxin is added to spin-labeled SN1, there is a reduction in normalized intensity of the EPR spectrum indicating a decrease in backbone motion (Fig. 6, *B* and *C*); the resulting lineshapes are consistent with those expected from a dynamic helical structure (45). Furthermore, the addition of syntaxin enhances the modulation depth at site 23R1 (Fig. 7*B*) and at other positions along SN1 (Figs. 7*E* and S12). These results are consistent with the formation of a 2:2 complex between SN1 and the SNARE motif of syntaxin ((19, 46), Fig. S10*A*), and the distances observed by DEER (Fig. 7*C*) are close to those predicted from the 2:2 crystal structure in Figure 7*A*. Addition of synaptobrevin to this 2:2 complex produces a slight increase in SN1 backbone ordering (Fig. 6*B*) and a significant decrease in the modulation depth (Fig. 7, *B* and *E*). This is consistent with the displacement of one of the SN1 fragments in the 2:2 complex by synaptobrevin, resulting in the formation of a 2:1:1 syntaxin:SN1:synaptobrevin complex. This complex is stable and can be purified by size-exclusion chromatography (Fig. S10*C*). Next, we repeated the previous experiments with labels placed at three different positions along synaptobrevin (Fig. 6*G*). Spectra from these labels are largely unchanged upon the addition of SN1 alone (Figs. 6*F* and S11*D*) but show evidence for assembly in the presence of SN1 and syntaxin. These results show that SN1, syntaxin, and synaptobrevin can strongly interact in the absence of SN2.

In the presence of syntaxin, SN2 behaves differently than SN1. There is no significant change in the normalized intensities along SN2 upon the addition of syntaxin, except near the N terminus at site 144 where there is a small but reproducible decrease in label motion (Figs. 6*D* and S11*B*). The C-terminal side of SN2 shows evidence for oligomerization, but unlike SN1, the addition of syntaxin reduces the modulation depth and oligomerization (Fig. 7*F*) rather than enhancing it. Unlike SN1, there is no evidence for the formation of a stable 2:2 complex between SN2 and syntaxin (Fig. S10*B*). When synaptobrevin was added to spin-labeled SN2 in the presence of syntaxin, no decreases in the backbone mobility (Fig. 6*D*) or significant changes in the modulation depth were observed (Fig. 7*F*), suggesting that SN2 remains unstructured when syntaxin and synaptobrevin are present. However, when spin labels on synaptobrevin are examined, there is evidence for an interaction with syntaxin and SN2 as seen by a decrease in normalized intensity in the EPR spectrum (Figs. 6*F* and S11*E*). Importantly, there are no significant changes in synaptobrevin label motion with the addition of isolated syntaxin, isolated SN1, or isolated SN2 (Fig. 6*F*). Moreover, size-exclusion chromatography of pre-incubated syntaxin-SN2-synaptobrevin resulted in two peaks eluting at different positions before the elution of the individual proteins (Fig. S10*D*), suggesting the presence of a weakly associated heterogeneous complex that may partially dissociate during the size-exclusion run.

In summary, our findings confirm and extend previous observations showing that the two SNARE motifs of SNAP25, SN1 and SN2, are not equivalent in their ability to interact with other SNAREs, with SN1 being able to form stable binary and ternary complexes with syntaxin alone, as well as with syntaxin and synaptobrevin, whereas SN2 shows only weak interactions with any of the partner SNAREs in the absence of SN1. The failure of SN2 to gain some clear secondary structure when present in a complex with syntaxin and synaptobrevin suggests that the SN2 segment may join the SNARE complex subsequent to SN1.

## Discussion

**Figure 8:**
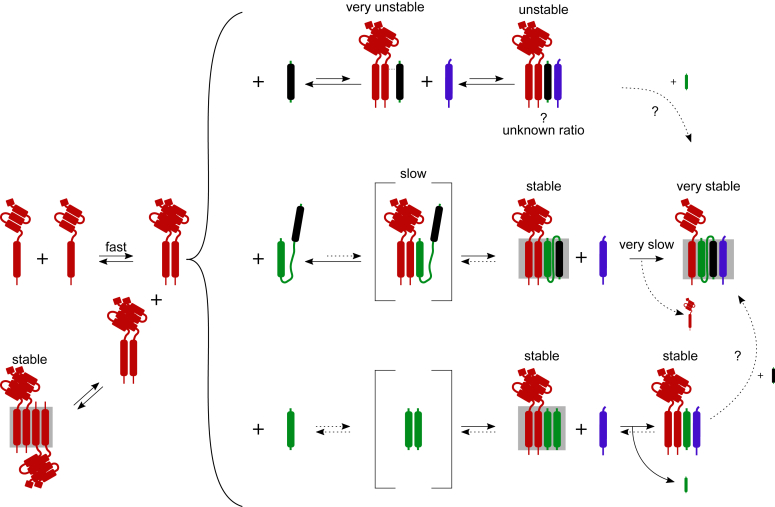
**Summary of the possible pathways for SNARE complex assembly.** Syntaxin monomers interact to form parallel dimers and antiparallel tetramers (crystal structure of the tetramer of syntaxin SNARE motif published in (25)). Syntaxin dimers are a prerequisite for interaction with SNAP25 and its motifs. The *middle* panel shows that interaction with full SNAP25 leads to the formation of 2:1 complex possibly through an intermediate of syntaxin dimer and an SN1 motif of SNAP25. 2:1 complex is reversible and stable, with structure resembling that of the SNARE complex (obtained using EPR and published in (17, 18, 34)). Synaptobrevin binds to this complex and forms the SNARE complex by displacing one of the syntaxins (crystal structure of the SNARE complex containing only SNARE domains published in Ref. (4) and including transmembrane regions of syntaxin and synaptobrevin published in (67)).The *lower* panel shows the pathway of assembly when SN1 motif is present instead of the full SNAP25. Syntaxin dimers form a stable 2:2 complex with SN1 (crystal structure of the SNARE motifs of the 2:2 complex published in (19)), possibly interacting with SN1 dimers. Synaptobrevin is able to bind to the 2:2 complex displacing one of the SN1 motifs and forming a stable 2:1:1 syntaxin:SN1:synaptobrevin complex. It is not known whether a SNARE complex can form from the 2:1:1 complex in the presence of SN2 motif. The *upper* panel shows that in the absence of SN1 motif of SNAP25, syntaxin and SN2 motif can form a very unstable transient complex that can be somewhat stabilized by the addition of synaptobrevin. Syntaxin:SN2:synaptobrevin complex is unstable, of unknown stoichiometry, and as in the case of 2:1:1 complex, it is unclear if it can transition to SNARE complex by addition of the SN1 motif. The only fast step in the entire pathway is the dimerization of syntaxin. The *gray rectangles* represent structures obtained in previous work; the *dashed arrows* and *brackets* represent steps that lack direct evidence.

In the present work, we have examined the reaction space of syntaxin (Qa), SNAP25 (Qb, Qc), and synaptobrevin (R) in solution using transient kinetics and EPR spectroscopy. While our findings confirm many of the previous reports, they highlight hitherto neglected features of the SNARE interaction landscape including, for instance, (i) syntaxin needs to dimerize before binding to SNAP25, with no evidence for a 1:1 intermediate; (ii) the 2:1 complex is an essential intermediate for full assembly in solution in the absence of regulatory proteins; (iii) the concentration of the 2:1 complex is low at physiological temperatures, (iv) synaptobrevin binding only occurs after association of syntaxin with one or both SNARE motifs of SNAP25; (v) syntaxin, synaptobrevin, and individual SN domains of SNAP25 form complexes of different stability that can be isolated; (vi) SN1 and SN2 motifs of SNAP25 join the SNARE complex at different time points, and (vii) with the exception of syntaxin dimerization, all other reactions, as far as we could measure them, proceed with slow kinetics, in agreement with earlier reports (7, 32). A cartoon showing the various intermediate states and the underlying association-dissociation reactions, integrating data from this work and previous publications, is shown in Figure 8.

Why are the binding equilibria of free SNAREs, measured here *in vitro* and in solution, important for the assembly pathway in presynaptic nerve terminals? As outlined in the Introduction, assembly of the neuronal SNAREs is regulated by four proteins that all bind to free SNAREs and/or to partially or fully assembled SNARE complexes. These include Munc18, Munc13, complexin, and synaptotagmin, each being represented by several isoforms. Of these, Munc18 and Munc13 are thought to guide initial assembly, whereas complexin and synaptotagmin are thought to act downstream of these proteins only after assembly has been initiated. However, these proteins associate only temporarily with the SNAREs. Moreover, in neurons, the SNAREs vastly outnumber each of them, in some cases by more than order of magnitude (*e.g.* Munc18 and Munc13, see (47)). Thus, the majority of all SNAREs are free to interact with each other most of the time. It is thus the mixture of monomers and various oligomers characterized here, which the regulatory proteins face as the starting point of assembly. This is particularly relevant for syntaxin and SNAP25 that colocalize at very high concentrations in the plasma membrane, with the equilibria likely shifted mostly towards the oligomeric states (syntaxin oligomers, 2:1 complexes).

There are presently two general models for how Munc18 initiates the formation of a metastable intermediate allowing for the initiation of SNARE zippering. In a first model, Munc18 chaperones an association between syntaxin and SNAP25, which functions as an acceptor template for the binding of synaptobrevin. In a second model, synaptobrevin and syntaxin are both aligned on the surface of Munc18 and form an acceptor template for SNAP25.

Both models invoke a primary interaction of Munc18 with syntaxin. Indeed, it was shown many years ago that Munc18 binds with high affinity to monomeric syntaxin in solution, stabilizing a “closed” conformation in which the Qa SNARE motif is in contact with an N-terminal regulatory domain (48). This interaction prevents oligomer formation or association with other SNAREs, and binding may even dissociate the 2:1 syntaxin:SNAP25 complex (49). It is still unclear whether the closed complex is an intermediate of the assembly pathway or represents a kinetic trap, with Munc13 being suggested to release syntaxin from this trap (*e.g.* reviewed in (50)). Intriguingly, Munc18 binding to syntaxin in native membranes containing SNAP25 is of much lower affinity (51). Indeed, there are indications that there are other binding modes for syntaxin and Munc18 (52) in which SNAP25 is associated with syntaxin in the complex (49). Moreover, previous work from one of our laboratories showed, both in solution and in native membranes, that Munc18 is capable of promoting the formation of a Q-SNARE acceptor complex capable of rapid and efficient binding of synaptobrevin (30, 53), supporting the first model. The findings reported here are compatible with this view as they show that there is no way synaptobrevin can bind before syntaxin and SNAP25 interact, with their SNARE motifs being aligned as template for binding. Accordingly, the role of Munc18 would be confined to avoiding the kinetic trap of the 2:1 complex by means of creating a reactive and metastable syntaxin–SNAP25 intermediate (probably 1:1, see (30, 49)). In this scenario, it remains to be established whether Munc18, in a first step, binds to these 2:1 complexes and then transforms them into a reactive intermediate, for example, by displacing one of the syntaxins or whether the pathway is different, for example, being initiated by Munc18 binding to syntaxin oligomers.

In support of the second model, a cryo-EM structure has recently been obtained showing both synaptobrevin and syntaxin bound to Munc18 (54). The structure supports the hypothesis that Munc18 may act to align both the R and Qa SNAREs for assembly with SNAP25 (29). However, the interaction between synaptobrevin and Munc18 is very weak (55, 56), and obtaining this structure required that synaptobrevin be cross-linked to the Qa-SNARE syntaxin. Considering that without cross-linking synaptobrevin does not bind to syntaxin in the absence of SNAP25, this structure does not address the question of whether the R-SNARE and Qa-SNARE assemble first onto Munc18 prior to SNAP25. It is conceivable that a weak affinity of Munc18 for synaptobrevin may function to increase the local concentration of synaptobrevin, thus bringing the R-SNARE into close proximity with an acceptor complex of syntaxin and SNAP25 for accelerating assembly of the SNARE complex.

The two SNARE-forming segments of SNAP25 (the Qb and Qc segments) are normally tethered together by a long linker that is palmitoylated and thus serves as membrane anchor (57). Although generally considered an internally disordered protein (58), high-resolution NMR spectroscopy showed that SN1 and SN2 part of SNAP25 have differential propensity for α-helix formation (59). Similar to these results, the EPR data presented here indicate that the two SNARE motifs are not equivalent in terms of their structure, dynamics, or ability to associate with syntaxin alone and both syntaxin and synaptobrevin. While SN1 forms a stable complex with syntaxin, with distances matching those of the high-resolution structure, the SN2 associates only weakly with syntaxin on its own, with its structure remaining disordered. This agrees with earlier observations that only SN1 segment interacts efficiently with syntaxin in cells (42), suggesting that initiation of the SNARE complex formation begins with syntaxin binding to SN1. The ability of syntaxin, SN1, and synaptobrevin to form a stable subcomplex raises the possibility that alignment of SN2 might be the final step in the assembly of the four-helix bundle.

In summary, when taking into consideration the high concentration of syntaxin and SNAP25 in the presynaptic membrane, it is likely that under steady-state conditions, most of syntaxin and SNAP25 are bound in oligomeric complexes that are in a kinetically trapped state. As far as structurally characterized (shaded boxes in Fig. 8), these states appear to represent rather stable four-helix bundles that require dissociation of at least one of the helices to proceed to the ternary SNARE complex, explaining the very slow kinetics of SNARE complex formation in the absence of the regulatory proteins. Biologically, this makes sense as it may be a safeguard mechanism preventing accidental “firing” of SNAREs upon contact with synaptobrevin-containing vesicles. Notably, synaptobrevin seems to be the only SNARE that is constitutively active ((60), but see (61) for an alternative view). The Q-SNARE oligomers may thus represent a large reserve pool that needs activation, which may involve disassembly by NSF. Activation allows the regulatory proteins to stabilize fusion-competent intermediates that otherwise are too unstable to be populated in the conformational landscape of the free SNAREs.

## Experimental procedures

### Protein constructs

The basic protein constructs used in this study were as folllows: syntaxin-1A (residues 1–265 and 1–250), SNAP25a (1–206), synaptobrevin2 (1–96), SN1 SNARE domain of SNAP25a (residues 1–83, and 7–83), and SN2 SNARE domain of SNAP25a (residues 120–206, and 141–204). All constructs were derived from *Rattus norvegicus*, and all were in a pET28a vector (7, 17), except for syntaxin-1A (1–265) which was in a pTXB1 vector (62). For stopped-flow experiments, single cysteine mutations were introduced in the following constructs to allow for maleimide fluorescent labeling: C225 for syntaxin-1A (1–265), C49 for SNAP25a (1–206), and C61 for synaptobrevin2 (1–96), while any natural occurring cysteine residues were mutated to serine residues. These mutations were previously shown not to interfere with the SNARE complex formation (17). Single cysteine mutations were also introduced for R1 spin labeling required for EPR experiments. The following residues were mutated to cysteine: E12, D23, E38, V48, R59, and D80 on SN1 (7–83); N144, G155, T173, S187, E194, and K201 on SN2 (141–204); S28, E55, and L93 on synaptobrevin-2 (1–96). Multiscale Modeling of Macromolecules (44) was used to predict rotamer populations in the assembled 4-helical bundle to ensure no rotamers disturbed the hydrophobic core of the SN1, syntaxin complex (PDB ID: 1JTH), or SNARE complex (PDB ID: 1SFC).

### Protein expression

Heat competent BL21(DE3) *Escherichia coli* cells were transformed with the corresponding protein construct and plated on an LB-agar plate supplemented with either kanamycin (pET28a) or ampicillin (pTXB1). A single colony was picked and grown primarily in LB and then TB media supplemented with salts (720 mM of K_2_HPO_4_ x 3H2O and 170 mM of KH_2_PO_4_) and the appropriate antibiotic. When the A_600_ reached between 0.8 and 1.0, the expression was initiated with 0.25 to 0.5 mM IPTG, and cultures were grown for additional 18 h at 18 °C or 22 °C. The cells were then pelleted at 9450*g* for 30 min at 4 °C and the pellet was washed with ice-cold PBS. For the *E. coli*–containing constructs used for EPR, the cells were grown only in LB medium and were pelleted at 9433*g* for 10 min at 4 °C without the PBS wash.

### Sample preparation for EPR

Pellets of syntaxin-1A (1–250), SN1 (7–83), SN2 (141–204), and synaptobrevin (1–96) were resuspended in lysis buffer (20 mM Hepes, 500 mM NaCl, 8 mM imidazole; pH ∼ 7.4) with added protease inhibitors leupeptin, AEBSF, and aprotinin at final concentrations 16.6 μg/ml, 66 μg/ml, and 28 KIU, respectively. 0.01 units/μl of benzonase nuclease was also added. The cells were lysed with five passes through a French press at a constant pressure of 16,000 psi. Urea was added to a final concentration of 2 M for SN1 and SN2 and 6 M for syntaxin and synaptobrevin. Syntaxin was additionally treated with 2% v/v of Triton X-100 and 1 mg of lysozyme. The samples were then incubated for 30 min at 4 °C and centrifuged at 36,000 rpm (Type 70 Ti rotor in Beckman Optima XPN Ultracentrifuge) for 1 h. The supernatant was afterward incubated with 2 ml of Ni^2+^-NTA beads per liter of protein at 4 °C for 2 h. The beads with bound synaptobrevin were washed with 250 column volumes (CV) of wash buffer (20 mM Hepes, 500 mM NaCl, and 20 mM imidazole; pH ∼ 7.4). The beads with bound SN1 or SN2 were washed with 50 CV of wash 1 buffer (20 mM Hepes, 750 mM NaCl, 20 mM imidazole, 2 M urea; pH ∼ 7.4), followed by 50 CV of wash 2 buffer (20 mM Hepes, 750 mM NaCl, 20 mM imidazole; pH ∼ 7.4). For syntaxin purification, four different buffers were used for washing, 50 CV each: wash 1 (20 mM Hepes, 500 mM NaCl, 20 mM imidazole, 6 M urea, 10% glycerol, 1% v/v Triton X-100; pH ∼ 7.4), wash 2 (20 mM Hepes, 500 mM NaCl, 20 mM imidazole, 20% glycerol, 1% v/v Triton X-100; pH ∼ 7.4), wash 3 (20 mM Hepes, 500 mM NaCl, 20 mM imidazole, and 1% v/v Triton X-100; pH∼7.4), wash 4 (20 mM Hepes, 500 mM NaCl, 20 mM imidazole, 0.1% w/v DPC; pH ∼ 7.4). SN1, SN2, and synaptobrevin were eluted with 25 CV of elution buffer (20 mM Hepes, 500 mM NaCl, 400 mM imidazole; pH ∼ 7.4), while the elution of syntaxin was done with 25 CV of the same elution buffer, but with the addition 0.1% w/v of DPC. All proteins were treated with 12.5 μg/ml of thrombin and incubated at 4 °C for 16 h and dialyzed or concentrated into 20 mM Hepes, 50 mM NaCl followed by 0.2 μm filtration. Anion exchange chromatography was performed on SN1 and SN2 (HiTrap Q HP – GE Healthcare) and cation exchange chromatography for synaptobrevin (HiTrap SP HP – GE Healthcare). A 0 mM-1000 mM NaCl gradient was used to elute the various proteins of interest for both ion-exchange methods. Size-exclusion chromatography was used to purify syntaxin (Superdex200 Increase 10/300 Gl – GE Healthcare) using 150 mM NaCl, 0.1% w/v DPC. The single cysteine variants of SN1, SN2, and synaptobrevin were all treated with 20-fold DTT for 2h at room temperature and ran on a Sephadex G-25 PD-10 desalting column (GE Healthcare). Immediately after, spin labeling was performed with 10-fold S-(1-oxyl-2,2,5,5-tetramethyl-2,5-dihydro-1H-pyrrol-3-yl) methyl methanesulfonothioate (Santa Cruz Biotechnology) at room temperature for 2 h. The PD-10 column was used again, and fractions containing labeled protein were collected. All proteins underwent extensive dialysis with 20 mM MOPS, 139 mM KCl, 12 mM NaCl buffer (pH ∼ 7.4). Syntaxin also contained 0.1% w/v DPC in the final buffer to keep the protein in its monomeric form (63).

### Sample preparation for stopped-flow and size-exclusion experiments

SN1 (1–83), SN2 (120–206), SNAP25a-C49 (1–206), and synaptobrevin2-C61 (1–96) were all purified in the same fashion. The bacterial pellet was resuspended in a lysis buffer (20 mM Hepes, 500 mM NaCl; pH ∼ 7.4) supplemented with DNase (2 μM final concentration; AppliChem GmbH), MgCl_2_ (1 mM final concentration), and protease inhibitors: trypsin inhibitor (0.5 mM final concentration; Sigma-Aldrich), benzamidine hydrochloride (10 mM final concentration; Sigma-Aldrich), phenylmethylsulfonyl fluoride (1 mM final concentration; Carl Roth GmbH). Breaking of the cells was done by sonication with a flat probe, at strength 8 and cycle set to 50% (Branson Sonifier W-450, Marshall Scientific). The cells were sonicated 6 times for 40 s with 40 s breaks on ice. The obtained homogenate was adjusted to 6 M urea, incubated for 15 min at room temperature with stirring, and then centrifuged for 1 h at 4 °C at 13,000 rpm (Fiberlite F14-6x250y Rotor in Sorvall RC 6+ Centrifuge; Thermo Fischer Scientific). Ni^2+^-NTA beads (3 ml of beads per 1 L of culture; His-Pur Ni-NTA, 88222, Thermo Fischer Scientific) were added to the supernatant, incubated for 3 h at 4 °C, and then washed with 15 CV of wash buffer (20 mM Hepes, 500 mM NaCl, 40 mM imidazole; pH ∼ 7.4). Proteins were eluted with 5 CV of elution buffer (20 mM Hepes, 500 mM NaCl, 400 mM imidazole; pH ∼ 7.4). Thrombin (3 μM final concentration; MP Biomedicals, LLC) and TCEP (0.1 mM final concentration) were added to the eluate, which was then dialyzed in dialysis buffer (20 mM Hepes, 150 mM NaCl, 1 mM EDTA, 0.1 mM TCEP; pH ∼ 7.4) at 4 °C for minimum of 12 h. After dialysis, the eluate was filtered with 0.2 μm filter (FP 30/0.2 CA-s, Whatman) and further purified using ion-exchange chromatography with a salt gradient of 0 mM to 1000 mM NaCl over 15 CV (for SNAP25a-C49, SN1 and SN2 anionic exchanger MonoQ 10/100 GL – Cytiva; for synaptobrevin cationic exchanger MonoS 10/100 GL – Cytiva).

As syntaxin-1A-C225 (1–265) was in pTXB1 vector which employs intein-CBD system (64), its purification differed slightly from previously described ones. After breaking of the cells by sonication, the homogenate was adjusted to 2 M urea. Following centrifugation, the supernatant was incubated with chitin beads (10 ml per 1 L of culture; S6651L, New England Biolabs) overnight at 4 °C with constant rotation (Test-tube-rotator 34528; Schütt Labortechnik). The beads were then washed with 25 CV of wash buffer and incubated overnight with 5 CV of elution buffer (see above). The subsequent procedure was the same as described above (filtering, dialysis, ion-exchange chromatography on anionic exchanger MonoQ 10/100 GL – Cytiva).

For stopped-flow experiments, single cysteine mutants were labeled with 5× molar excess of either Alexa Fluor 488 C5 maleimide (donor dye; A10254, Thermo Fischer Scientific) or Alexa Fluor 647 C2 maleimide (acceptor dye; A20347, Thermo Fischer Scientific). The dye powder was dissolved in DMSO, added to the proteins, and incubated overnight at 4 °C on a rotator (Test-tube-rotator 34528; Schütt Labortechnik) and protected from light. The labeled protein was separated from the free dye using size-exclusion chromatography (Superdex75 10/300 FPLC – 9738031, Pharmacia Biotech) in the experiment buffer (20 mM Hepes, 150 mM KCl, 0.1 mM TCEP; pH ∼ 7.4).

### Stopped-flow measurements

All stopped-flow measurements were performed in a two-syringe mode on a SX20D stopped-flow spectrophotometer (Applied Photophysics) at 37 °C (unless stated otherwise; Ecoline staredition RE104, Lauda Dr R. Wobser GmbH & Co KG). The donor dye was excited with SX LED Light Source PSU (Applied Photophysics) set to 10 mA and 470 nm optical cable (Applied Photophysics), while the emission from the acceptor dye was monitored using a near-infrared PMT 300 to 900 nm (Type R2228, Applied Photophysics) with 665 nm cut-on filter (RG665, Schott). Before acquisition of data, the recording cell was primed with four triggering events. Two technical replicates (traces) were recorded for every condition with 10, 000 datapoints per trace. Every trace was recorded in a logarithmic time scale with disabled oversampling. Fitting of the raw traces was done using either KinTek Explorer (Professional version 6.3; KinTek Corporation) or the OriginPro (2019b; OriginLab Corporation) software. The goodness of the fit was assessed by the distribution of the residuals, the confidence intervals of the measured parameters, and the χ^2^/DoF (KinTek Explorer) or R^2^ (OriginPro) value. Apparent rate constants (kobs) obtained from the fitted raw traces were averaged and further fitted to the linear or hyperbolic equation as indicated (see Results).

### Size-exclusion experiments

Prior to the size-exclusion experiments, the ion-exchange fractions were run through a preparatory size exclusion for further purification (Superdex75 10/300 FPLC – 9738031, Pharmacia Biotech) and buffer exchange to experiment buffer (20 mM Hepes, 150 mM KCl, 0.1 mM TCEP; pH ∼ 7.4). For every protein, the peak fraction was selected and mixed with other proteins in different combinations (see Results) in a 1:1 ratio (final concentration of 5 μM for each protein and 600 μl of total volume). The combined proteins were incubated overnight on a rotator (Test-tube-rotator 34528; Schütt labortechnik) and prior to loading, centrifuged for 10 min at 4 °C and 14,000 rpm (Fresco 21 Microcentrifuge; Heraeus; Thermo Fischer Scientific) to remove any aggregates. The samples were then run on a Superdex200 Increase 10/300 column (28-9909-44; Cytiva) in experiment buffer (see above) at 4 °C. Peak fractions collected during the size-exclusion run were separated by SDS-PAGE and visualized with Coomassie staining. The gels were subsequently scanned using Epson Perfection V850 Pro scanner and Epson Scan software in Professional Mode (Film (with Film Area Guide), Positive Film, 24 bit color, 350 dpi).

### CW EPR measurements

For all continuous wave EPR experiments, 6 μl of sample was loaded into borosilicate capillaries (VitroCom) with an inner diameter of 0.6 mm and an outer diameter of 0.84 mm. Each sample contained 30% sucrose to slow the rotational diffusion of the protein so that the EPR spectra reflect local changes in protein structure of spin-labeled side-chain contact rather than overall protein diffusion. The nitroxide spectra were collected with a Bruker EMX spectrometer operating at X-band, using an ER 4123D dielectric resonator (Bruker Biospin). Each experiment was performed at room temperature using 2 mW incident microwave power, 1 G field modulation, and a 100 G field sweep. The spectra were normalized, phased, and plotted using in-house programs written by David Nyenhuis. The normalized intensities (A_pp_) provide a measure of the mobility of the spin label (65), and in the case of solvent-exposed sites, they reflect protein backbone motion on the ns time-scale (66). Estimates of rotational correlation times for the nitroxide side chain were made using LabVIEW software provided by Wayne Hubbell and Christian Altenbach (UCLA).

### DEER measurements

For all DEER experiments, 20 μl of sample was loaded into quartz capillaries (VitroCom) with an inner diameter of 1.1 mm and an outer diameter of 1.6 mm. Each sample contained 20% deuterated glycerol and were flash frozen using liquid nitrogen. Spectra were collected using a Bruker Elexsys E580 spectrometer operating at Q-band with an EN5107D2 dielectric resonator (Bruker Biospin) and a 300 W TWT Amplifier (Applied Systems Engineering). Each experiment was performed at 50 K using a dead time-free four-pulse DEER sequence. Rectangular pulses were used with lengths of π/2 = 10 ns and π = 20 ns. The probe and pump frequencies were at a 75 MHz offset. The dipolar time evolution data was processed with Tikhonov regularization or the DEERNet routine using the software package DeerAnalysis2022 (44).

## Data availability statement

All data is contained within the manuscript. The raw data (excel sheets from recordings) will be shared by the corresponding author upon request.

## Supporting information

This article contains supporting information.

## Conflicts of interest

The authors declare that they have no conflicts of interest with the contents of this article.
