# Supplementary material for: Intermediate steps in the formation of neuronal SNARE complexes
Source: J Biol Chem. 2024 Jul 19;300(8):107591. doi: 10.1016/j.jbc.2024.107591 (PMC11381810; doi:10.1016/j.jbc.2024.107591)
Supplement: Supporting information [file mmc1.pdf]

Supporting Information

Intermediate steps in the formation of neuronal SNARE complexes

Sonja Pribicevic[1]†, Abigail C. Graham[2]†, David S. Cafiso[2], Ángel Pérez-Lara[1,3], Reinhard Jahn[1]

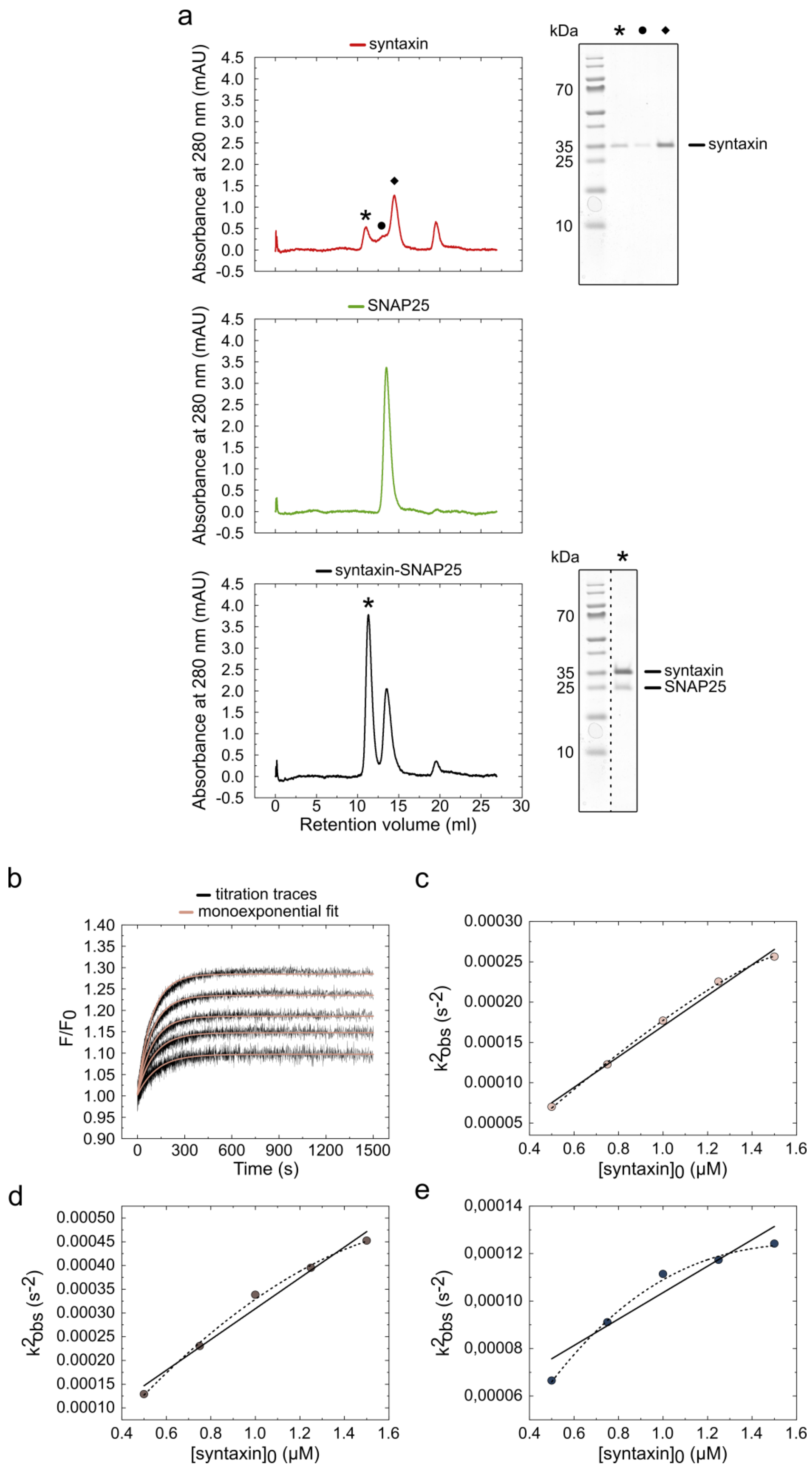
a
syntaxin
Absorbance at 280 nm (mAU)
kDa
70
35
25
10
syntaxin
SNAP25
syntaxin-SNAP25
Retention volume (ml)
b
titration traces
monoexponential fit
F/F0
Time (s)
c
k2obs (s-2)
[syntaxin]0 (μM)
d
e

Figure S1. Syntaxin and SNAP25 form a 2:1 complex. *(a) Elution profiles of 5 µM of syntaxin (red curve, upper graph), SNAP25 (green curve, middle graph) and the syntaxin-SNAP25 complex (black curve, bottom graph). The samples were primarily incubated overnight at 4°C, which was followed by size exclusion chromatography at the same temperature. The elution profile of syntaxin shows oligomer formation with three distinguishable peaks at approximately 11 ml, 13.2 ml and 14.5 ml, that presumably correspond to syntaxin tetramer, dimer and monomer, respectively. The elution profile of the syntaxin-SNAP25 sample contains free SNAP25 (unmarked peak in the bottom graph), but no free syntaxin, consistent with the formation of the 2:1 complex. The relevant peaks (indicated with symbols) were analyzed by Coomassie stained SDS-PAGE and are shown next to the corresponding chromatogram. The original image was converted to a greyscale image. Both SDS-PAGE images originate from the same gel and contain the same gel ladder, with the bottom image being cropped as indicated by the dotted line. The top SDS-PAGE image shows the ladder and lines 1-3, while the bottom SDS-PAGE image shows again the ladder and line 11 of the same gel. (b) Equimolar titration traces of syntaxin and SNAP25 were fit to a monoexponential equation:* $y = Ae^{(-k_{obs}x)} + C$; *where* y *corresponds to the fluorescence intensity,* A *to the fluorescence amplitude,* x *to the time,* C *to the starting point of the fit, and* k_{obs} *to the observed rate constant. (c, d, e) Plots of the square of the apparent rate constant (*k_{obs}^2*) and the initial concentration of syntaxin (*$[\text{syntaxin}]_0$*) for every experiment can be best fit with a hyperbolic (dashed curves), rather than a linear function (full line), indicating that syntaxin and SNAP25 binding is not in 1:1 stoichiometry and thus does not occur in a single step reaction. Fitting was performed using the Generalized Hyperbola function (OriginPro 2019b, OriginLab Corporation):* $y = a - \left(b/(1+cx)^{1/d}\right)$ *; where y corresponds to fluorescence intensity, x to the initial syntaxin concentration, while a, b, c, and d are the parameters determining the shape of the hyperbola: a corresponds to the horizontal asymptote; for d>0 hyperbola has a vertical asymptote equal to* $\frac{1}{c}$*, for d<0 hyperbola has an oblique asymptote with the slope of (*$b \times c$*) and an intercept of (*$a - b$*).*

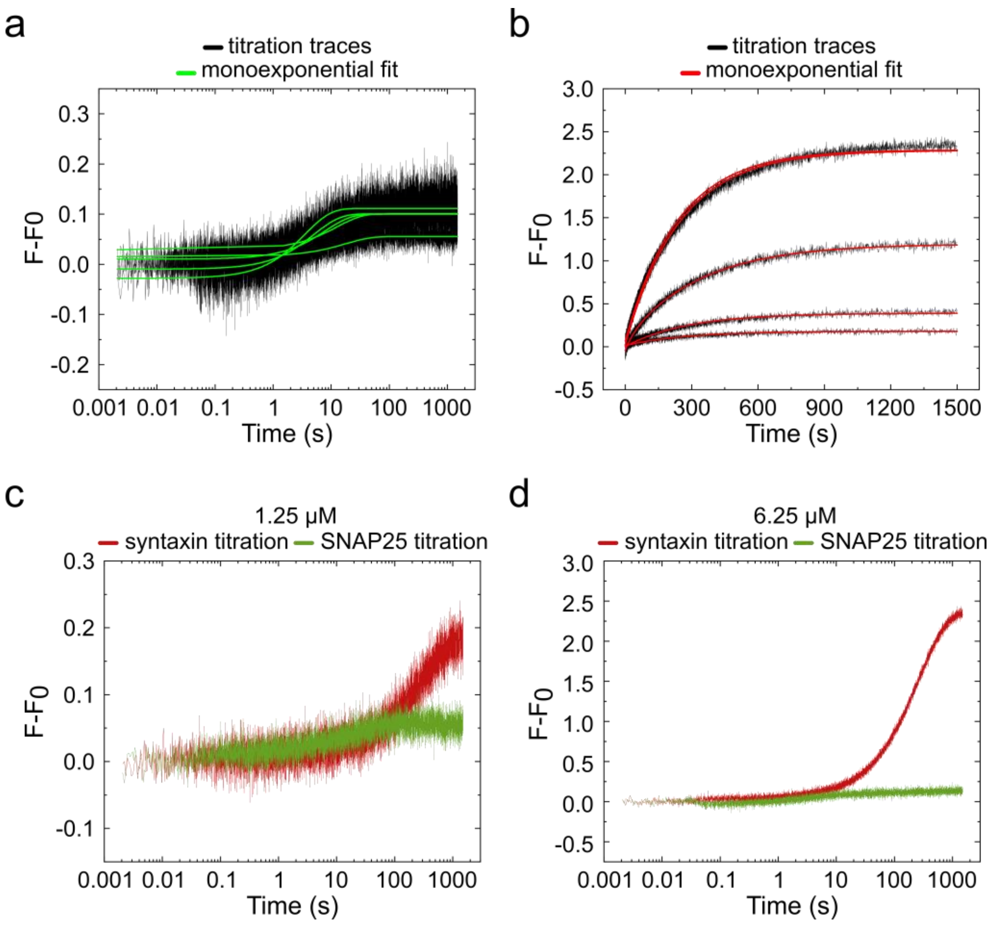

Figure S2. Low syntaxin dimer concentration leads to lower fluorescence amplitudes and therefore lower 2:1 complex formation. *(a) Monoexponential fit of the traces from SNAP25 titration over syntaxin experiment and (b) syntaxin titration over SNAP25 experiment. The traces were fit to the following equation:* $y = Ae^{(-k_{obs}x)} + C$ *(see above). Comparison between syntaxin and SNAP25 titrations show similar noise level at the lowest (c) and highest (d) titrant concentrations. In the case of SNAP25 titration (green traces), the low concentrations of syntaxin dimer causes the reaction to reach only a fraction of the amplitude achieved with syntaxin titration (red traces).*

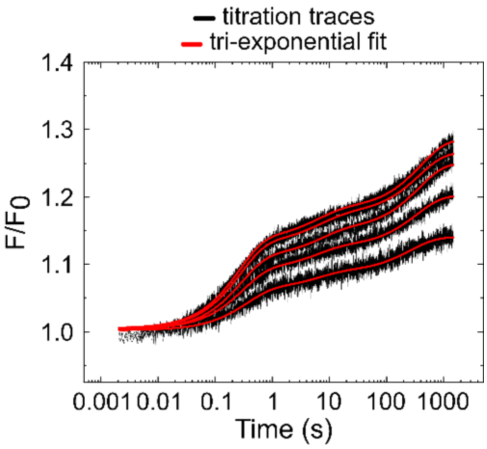

Figure S3. Fitted titration traces showing syntaxin oligomerization. *Titration traces from experiment testing syntaxin-syntaxin interaction (shown in Figure 3a) were successfully fitted to a tri-exponential equation:* $y = A_1e^{(-k_{1obs}x)} + A_2e^{(-k_{2obs}x)} + A_3e^{(-k_{3obs}x)} + C$ *(see above).*

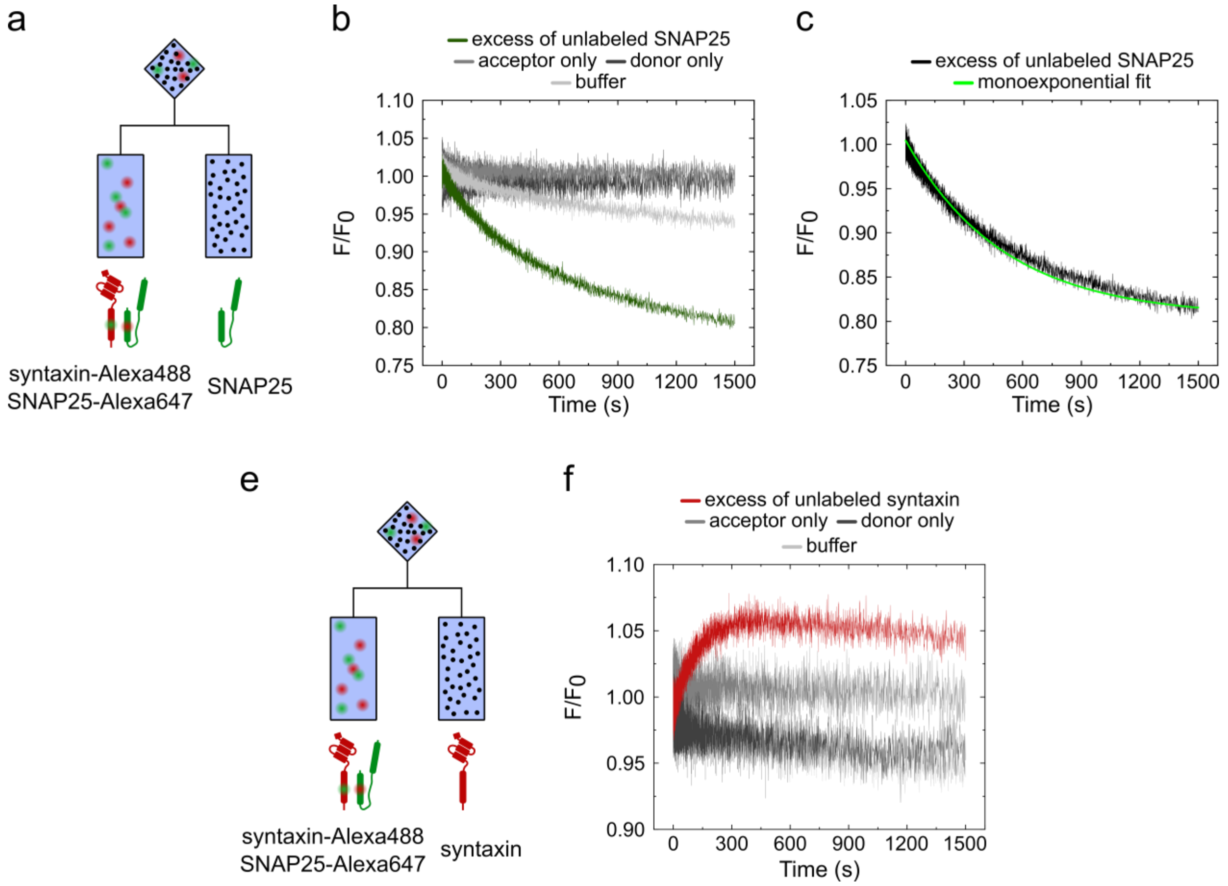

Figure S4. Addition of a 10-fold excess of unlabeled syntaxin or SNAP25 to labeled syntaxin and SNAP25 at equilibrium yield opposite FRET changes that are due to the low starting concentration of the 2:1 complex. *(a) Donor-labeled syntaxin (green dots) and acceptor-labeled SNAP25 (red dots) were incubated at equimolar concentrations to form a 2:1 complex. After equilibration (approx. 10 min), unlabeled SNAP25 was added and the change in the acceptor fluorescence was monitored over time. Final concentrations were 1 µM for the labeled proteins and 10 µM for unlabeled SNAP25. (b) Addition of unlabeled SNAP25 caused a decrease in the acceptor fluorescence as the labeled SNAP25 in the 2:1 complex is replaced with unlabeled SNAP25 (green trace). The slight decrease observed in the buffer control is caused by dissociation due to dilution of the complex. (c) Mono-exponential fit (see above) of the dissociation yielded a* k_{off} *of (3.44 ± 0,02) x* 10^{-3} s^{-1}*. (e) Same as in a) but this time excess unlabeled syntaxin (7.5 µM final concentration) was added to donor-labeled syntaxin (0.75 µM, green dots) and acceptor-labeled SNAP25 (0.75 µM, red dots). (f) In contrast to b), an increase rather than a decrease of the acceptor fluorescence (red trace) was observed that lasted ~600 s, and was absent from controls, indicating that for saturating SNAP25 in the 2:1 complex much higher concentrations of syntaxin are required.*

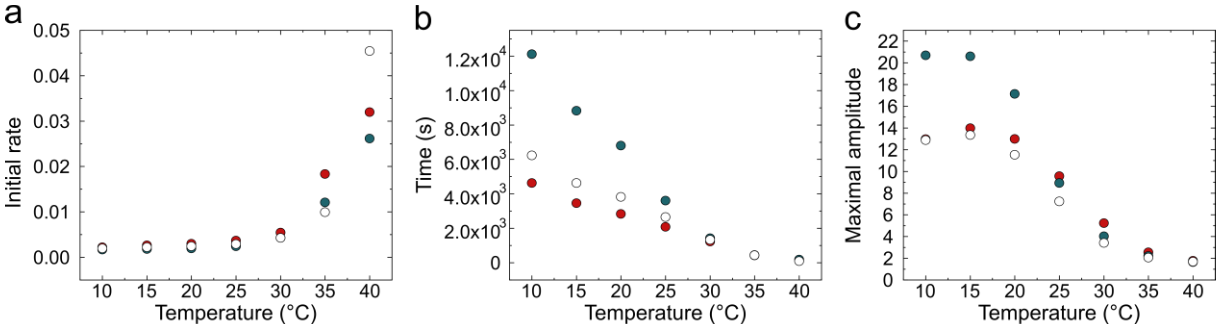

Supplementary Figure 5. 2:1 complex formation at different temperatures. *(a) Initial rate (first derivative of the normalized fluorescence intensity of the fitted trace at time point t=0.002 s) of the 2:1 complex formation increases with the increasing temperature indicating faster rate at a higher temperature. (b) The time it takes to reach the plateau decreases with the increasing temperature indicating that the steady-state is reached faster at higher temperature. (c) The change of the maximal amplitude of the acceptor fluorescence signal with temperature shows that at high temperatures, the 2:1 complex concentration is low and vice versa. Three different colors (red, white and blue) represent three repetitions of the experiment.*

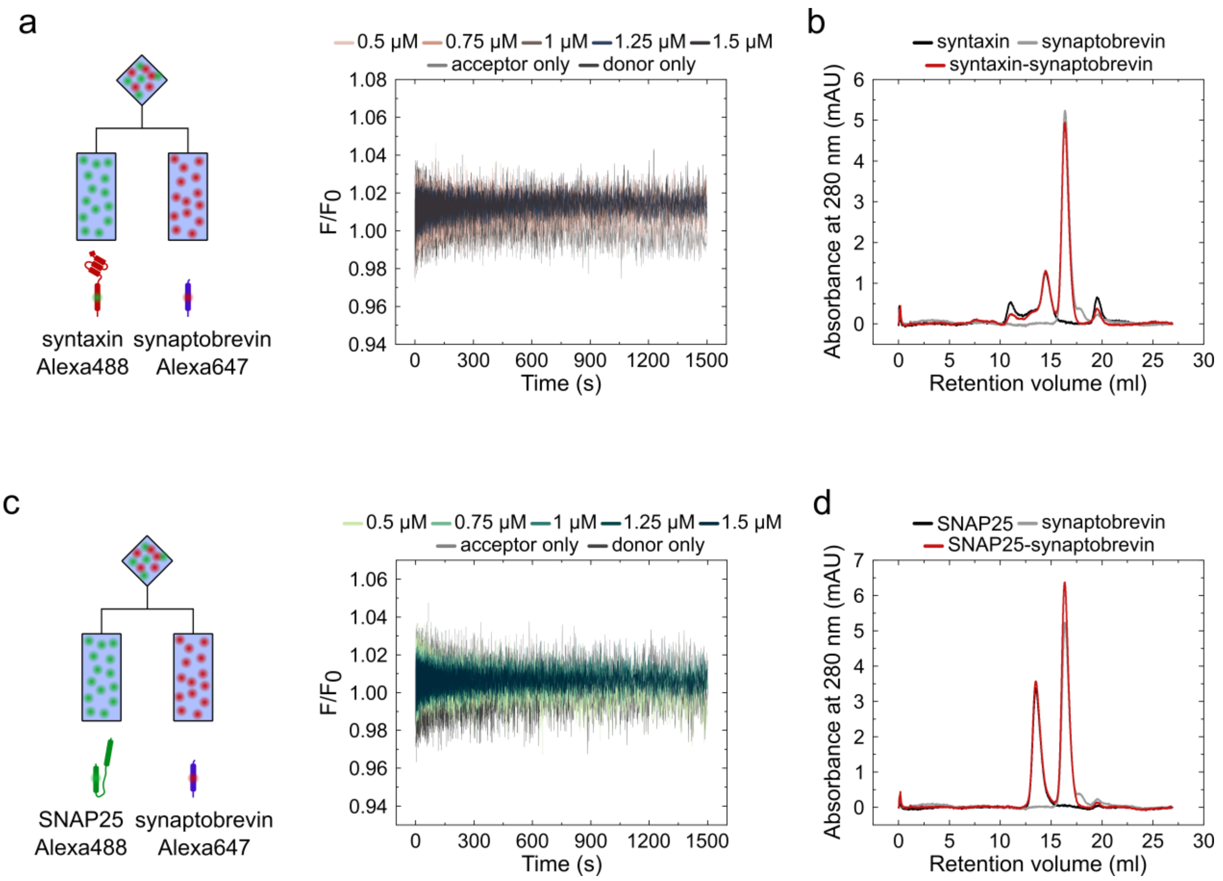

Figure S6. Synaptobrevin does not form complexes with syntaxin, or SNAP25. *(a) Schematic representation of equimolar titrations of donor-labeled syntaxin and acceptor-labeled synaptobrevin, and (c) donor-labeled SNAP25 and acceptor-labeled synaptobrevin. Upon triggering, equal volumes of the labeled proteins was mixed and the interaction was monitored in the recording cell. Titration traces of both experiments show no difference to the control even at the highest concentrations of 1.5 μM, showing that synaptobrevin does not interact with either SNAP25 or syntaxin alone. (b, d) Size exclusion chromatography of syntaxin-synaptobrevin, and SNAP25-synaptobrevin (5 μM each), respectively, after incubation overnight at 4°C, showed only superimposition of the free monomer elution profiles, confirming the absence of heterooligomeric complexes.*

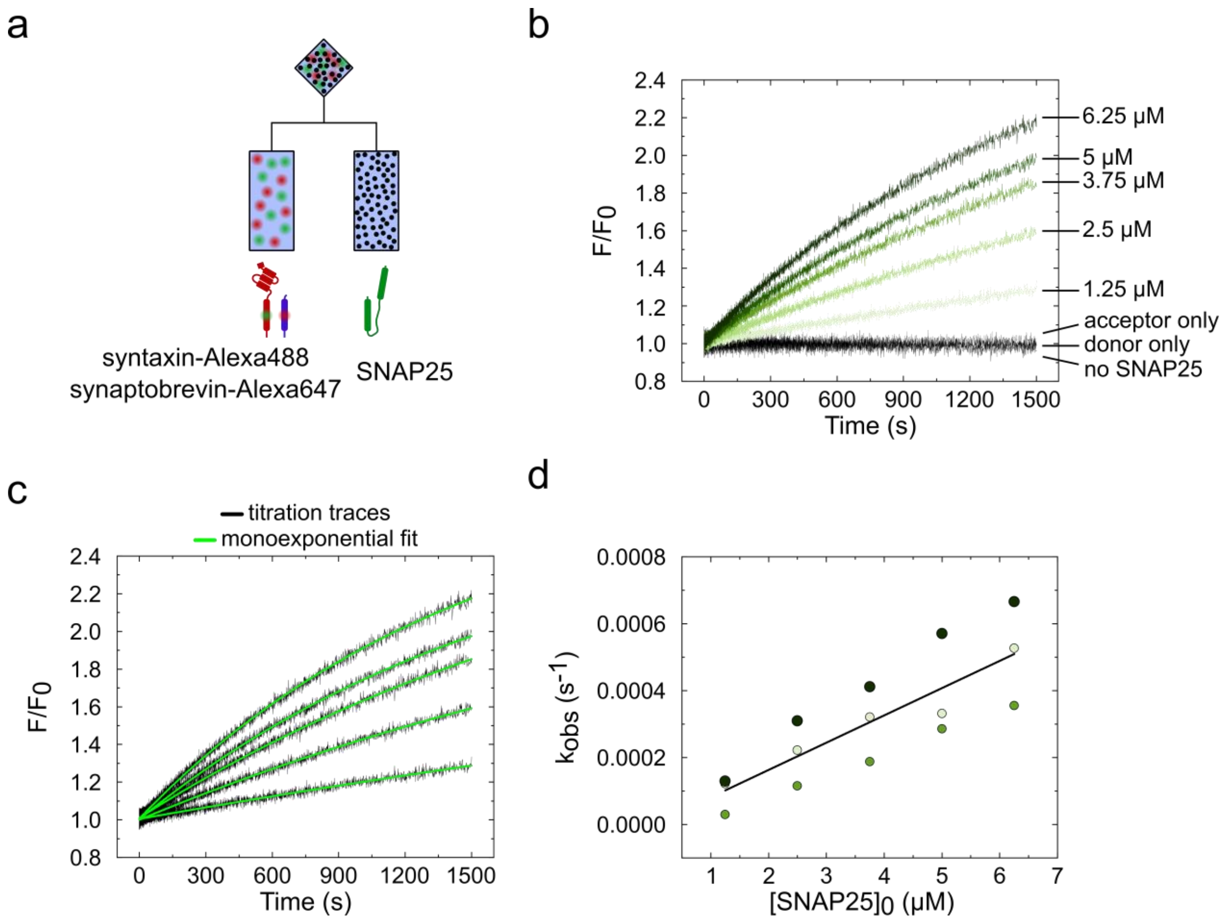

Figure S7. SNARE complex formation in the presence of excess SNAP25 can be fit to a monoexponential function. *(a) Excess of unlabeled SNAP25 was added to premixed donor-labeled syntaxin and acceptor-labeled synaptobrevin (b) titration traces showed an increase that corresponded to the increase in the concentration of SNAP25, while all the controls remained flat. (c) The traces were best fit to a monoexponential equation ($y = Ae^{(-k_{obs}x)} + C$, see above) and (d) the obtained k_{obs} showed a linear increase with increasing SNAP25 concentrations. The three different shades of green (d) correspond to three repetitions of the experiment.*

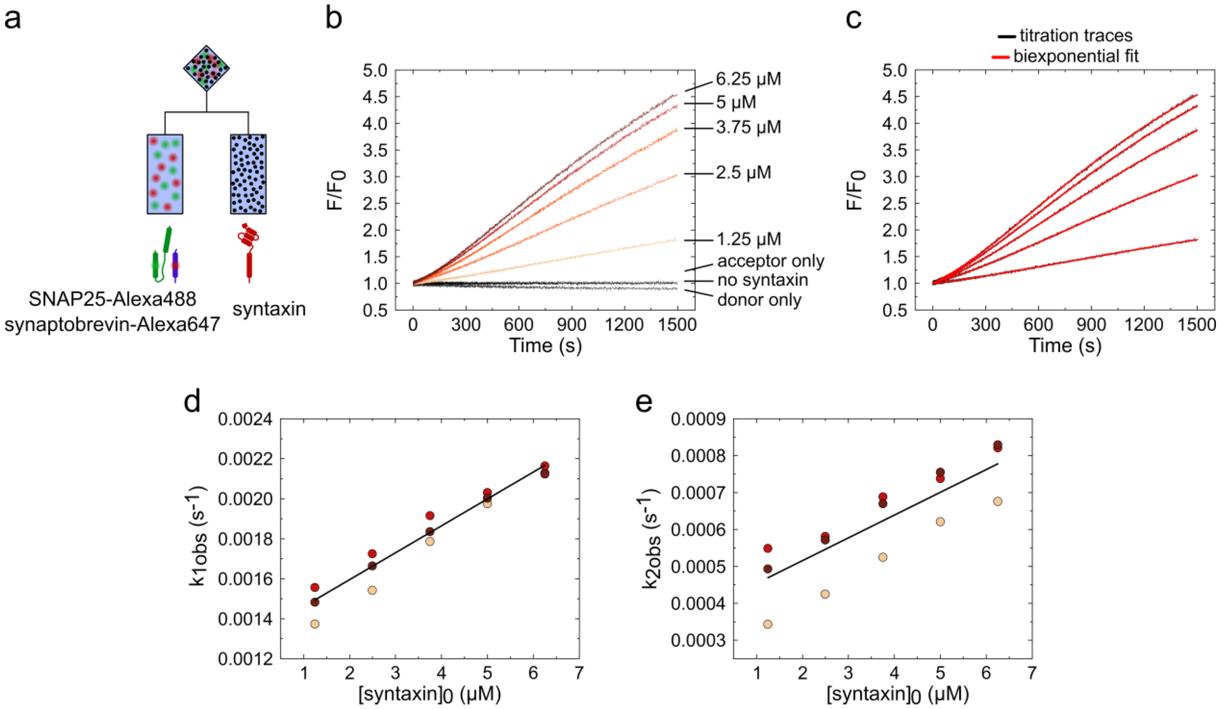

Figure S8. SNARE complex formation in the presence of excess of syntaxin allows for capturing of two kinetically distinct phases. *(a) Excess of unlabeled syntaxin was titrated over pre-mixed donor-labeled SNAP25 and acceptor-labeled synaptobrevin. (b) The titration traces show a robust increase that was absent from all controls. (c) Two phases could be distinguished in the titration traces that were then fit to a biexponential equation ($y = A_1 e^{(-k_{obs_1}x)} + A_2 e^{(-k_{obs_2}x)} + C$, see above), yielding k_{1obs} and k_{2obs}. (d, e) Both detected phases showed linear dependence of the initial syntaxin concentration with the first phase (described by k_{1obs}) being compatible with binding of SNAP25 to syntaxin dimer as shown in main text in Figure 2c, and the second phase compatible to the SNARE complex formation. The three different colors (d, e) correspond to three repetitions of the experiment.*

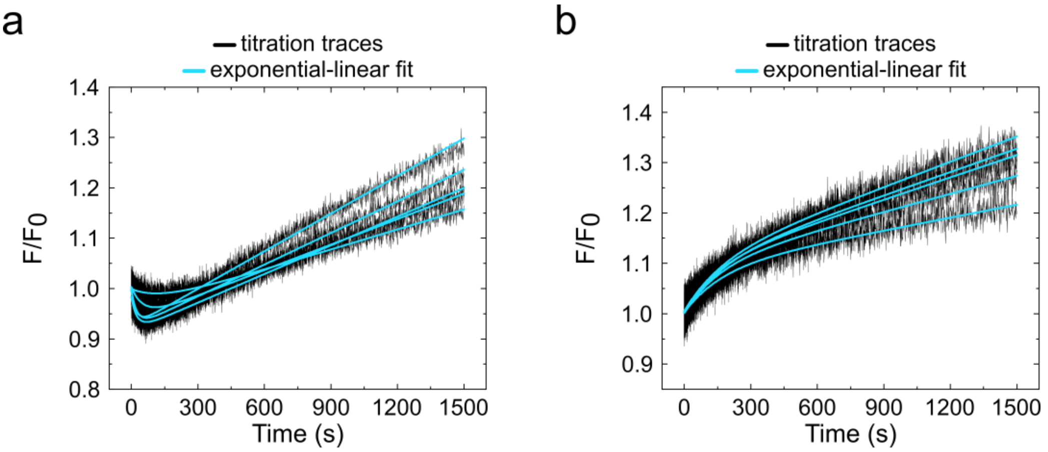

Figure S9. Fitted synaptobrevin titration traces. *For both experiments, the titration traces show two clearly distinguishable phases that were successfully fit to an exponential-linear equation (blue trace):* $y = A_1 e^{(-k_{obs_1} x)} + k_{obs_2} x + C$ *(see above).*

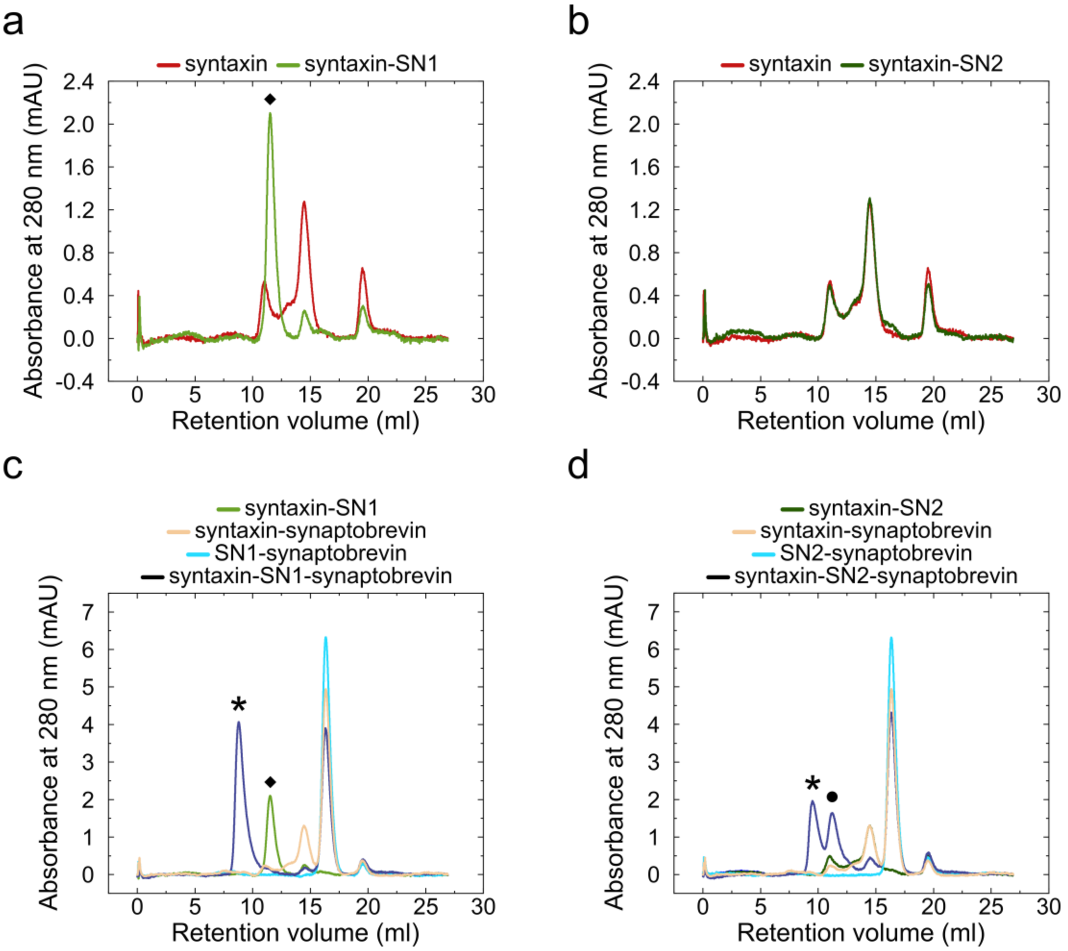

Figure S10. Analysis of SNARE sub-complexes using size-exclusion chromatography following an overnight incubation of SNARE mixtures in comparison to free SNAREs. *(a, c) Syntaxin and SN1 alone and with synaptobrevin show formation of a stable complex as indicated by sharp peaks in the chromatograms eluting at ~11.6 ml (marked with a diamond) and ~8.8 ml (marked with a star), respectively. (b, d) While there was no indication of a complex formation between syntaxin and SN2, addition of synaptobrevin yielded two peaks in the chromatogram at ~9.5 ml (marked with a star) and ~11.2 ml (marked with a circle), consistent with heterogeneous and unstable complex that likely dissociates during the size exclusion run.*

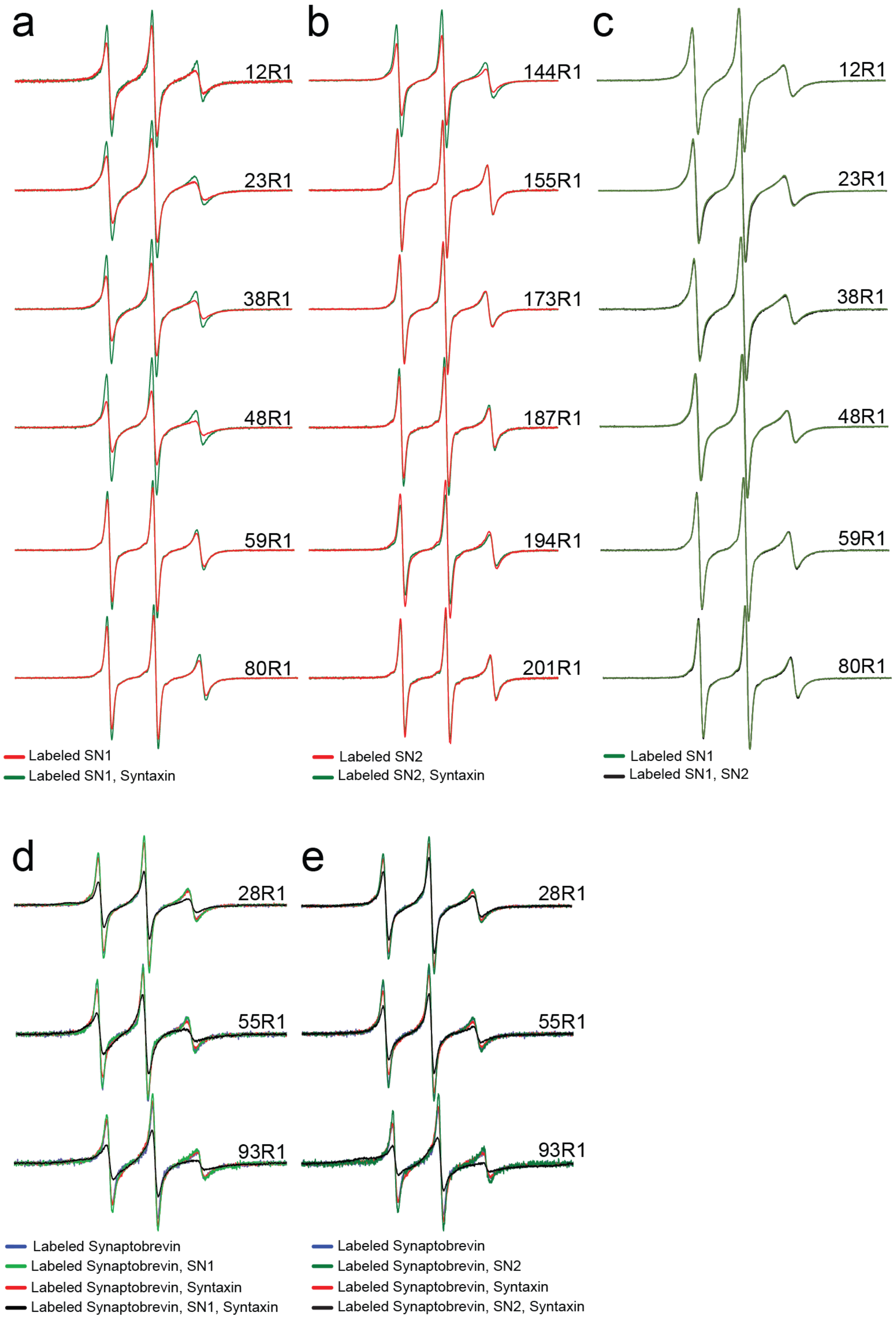

Figure S11. Continuous Wave Electron Paramagnetic Resonance Analysis. (a) *Normalized X-band EPR spectra of single SNAP25 SN1 (7-83) labels in the absence (green) and presence of syntaxin (1-250) (red). Each of the six single-labeled SN1 fragments are at a final concentration of 24 μM and syntaxin is at 32 μM. (b) Normalized X-band EPR spectra of single SNAP25 SN2 labels in the absence*

(green) and presence of syntaxin (1-250) (red). Each of the six single-labeled SN2 fragments are at a final concentration of 24 μM and syntaxin is at 32 μM. (c) Normalized X-band EPR spectra of 24 μM SNAP25 SN1 (7-83) labels in the absence (green) and presence (black) of 24 μM SN2 (141-204) (d) Normalized X-band EPR spectra of labeled synaptobrevin in various conditions. 25 μM isolated synaptobrevin is shown in blue. 25 μM synaptobrevin in the presence of 28 μM syntaxin is in red. 25 μM synaptobrevin in the presence of 28 μM SN1 is in green. 25 μM synaptobrevin in the presence of 28 μM SN1 and 28 μM syntaxin is in gray. 28R1 and 93R1 synaptobrevin, and the added proteins follow the concentration scheme described above. 55R1 synaptobrevin is at 26 μM in all four conditions. When SN1 and syntaxin are added to 55R1 synaptobrevin, both proteins are at 30 μM. (e) Normalized X-band EPR spectra of labeled synaptobrevin in various conditions. 25 μM isolated synaptobrevin is shown in blue. 25 μM synaptobrevin in the presence of 28 μM syntaxin is in red. 25 μM synaptobrevin in the presence of 28 μM SN2 is in green. 25 μM synaptobrevin in the presence of 28 μM SN2 and 28 μM syntaxin is in black. 28R1 and 93R1 synaptobrevin, and the added proteins follow the concentration scheme described above. 55R1 synaptobrevin is at 26 μM in all four conditions. When SN2 and syntaxin are added to 55R1 synaptobrevin, both proteins are at 30 μM.

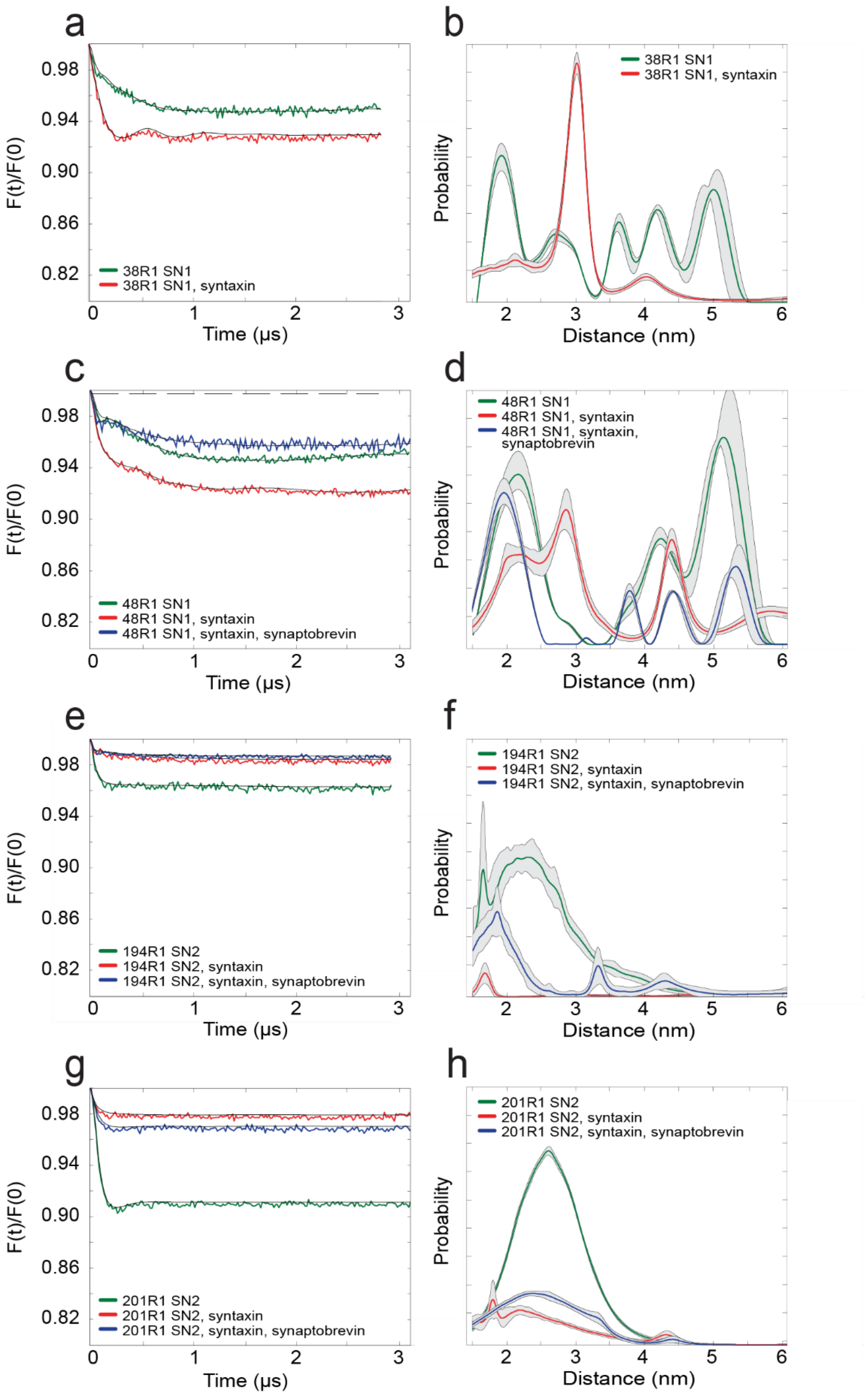

Figure S12. Double Electron-Electron Resonance Analysis. (a) *Background-corrected DEER data obtained for 24 μM 38R1 SN1 (green trace), 24 μM 38R1 SN1 in the presence of 24 μM syntaxin (red trace). (b) Distance distributions for 24 μM 38R1 SN1 (green), 24 μM 38R1 SN1 in the presence of 24 μM Syntaxin (red).* (c) *Background-corrected DEER data obtained for 30 μM 48R1 SN1 (green trace), 30 μM 48R1 SN1 in the presence of 33 μM Syntaxin (red trace), 30 μM 48R1 SN1 in the presence of 33*

μM syntaxin and 33 μM synaptobrevin (blue trace). (d) Distance distributions for 30 μM 48R1 SN1 (green), 30 μM 48R1 SN1 in the presence of 33 μM syntaxin (red), and 30 μM 48R1 SN1 in the presence of 33 μM syntaxin and 33 μM synaptobrevin (blue trace). (e) *Background-corrected DEER data obtained for 29 μM 194R1 SN2 (green trace), 29 μM 194R1 SN2 in the presence of 29 μM syntaxin (red trace), 29 μM 194R1 SN2 in the presence of 29 μM syntaxin and 29 μM synaptobrevin (blue trace). (f) Distance distributions for 29 μM 194R1 SN2 (green), 29 μM 194R1 SN2 in the presence of 29 μM syntaxin (red), and 29 μM 194R1 SN2 in the presence of 29 μM syntaxin and 29 μM synaptobrevin (blue trace).* (g) *Background-corrected DEER data obtained for 29 μM 201R1 SN2 (green trace), 29 μM 201R1 SN2 in the presence of 29 μM Syntaxin (red trace), 29 μM 201R1 SN2 in the presence of 29 μM syntaxin and 29 μM synaptobrevin (blue trace). (h) Distance distributions for 29 μM 201R1 SN2 (green), 29 μM 201R1 SN2 in the presence of 29 μM syntaxin (red), and 29 μM 201R1 SN2 in the presence of 29 μM syntaxin and 29 μM synaptobrevin (blue trace).*
